# Observing Convective Aggregation

**DOI:** 10.1007/s10712-017-9419-1

**Published:** 2017-06-28

**Authors:** Christopher E. Holloway, Allison A. Wing, Sandrine Bony, Caroline Muller, Hirohiko Masunaga, Tristan S. L’Ecuyer, David D. Turner, Paquita Zuidema

**Affiliations:** 1 Department of Meteorology, University of Reading, Reading, RG6 6BB UK; 2 Lamont-Doherty Earth Observatory, Columbia University, PO Box 1000, 61 Route 9W, Palisades, NY 10964-1000 USA; 3 Department of Earth, Ocean and Atmospheric Science, Florida State University, Mail Code 4520, PO Box 3064520, Tallahassee, FL 32306-4520 USA; 4 Sorbonne University, LMD/IPSL, CNRS, Univ Paris 06, mailbox 99, 4 Place Jussieu, 75252 Paris cedex 05, France; 5 LMD/IPSL, CNRS, École Normale Supérieure, Paris Sciences Et Lettres, 24 rue Lhomond, 75230 Paris cedex 05, France; 6 Institute for Space-Earth Environmental Research, Nagoya University, Furo-cho, Chikusa-ku, Nagoya, 464-8601 Japan; 7 Department of Atmospheric and Oceanic Sciences, University of Wisconsin-Madison, 1225 West Dayton Street, Madison, WI 53706 USA; 8 NOAA/Earth System Research Laboratory, Global Systems Division, 325 Broadway, Boulder, CO 80305-3337 USA; 9 Rosenstiel School of Marine and Atmospheric Science, University of Miami, 4600 Rickenbacker Causeway, Miami, FL 33149 USA

**Keywords:** Self-aggregation, Tropical convection, Convective organization, Climate sensitivity, Cloud feedback

## Abstract

Convective self-aggregation, the spontaneous organization of initially scattered convection into isolated convective clusters despite spatially homogeneous boundary conditions and forcing, was first recognized and studied in idealized numerical simulations. While there is a rich history of observational work on convective clustering and organization, there have been only a few studies that have analyzed observations to look specifically for processes related to self-aggregation in models. Here we review observational work in both of these categories and motivate the need for more of this work. We acknowledge that self-aggregation may appear to be far-removed from observed convective organization in terms of time scales, initial conditions, initiation processes, and mean state extremes, but we argue that these differences vary greatly across the diverse range of model simulations in the literature and that these comparisons are already offering important insights into real tropical phenomena. Some preliminary new findings are presented, including results showing that a self-aggregation simulation with square geometry has too broad distribution of humidity and is too dry in the driest regions when compared with radiosonde records from Nauru, while an elongated channel simulation has realistic representations of atmospheric humidity and its variability. We discuss recent work increasing our understanding of how organized convection and climate change may interact, and how model discrepancies related to this question are prompting interest in observational comparisons. We also propose possible future directions for observational work related to convective aggregation, including novel satellite approaches and a ground-based observational network.

## Introduction

From the very first studies describing convective self-aggregation (e.g., Held et al. 1993; Tompkins 2001; Bretherton et al. 2005), the spontaneous clustering of convection, cloud, and moisture in idealized numerical simulations of radiative–convective equilibrium (RCE) despite homogeneous initial conditions, boundary conditions, and forcing (cf. Wing et al. 2017), there has been a recurring question: Is this “real”? In other words, is the intriguing clumping behavior representative of actual convective organization in nature, or is it just a model artifact? And, to the extent that the behavior is relevant for understanding real atmospheric convection, what does it tell us about the role of convective organization in weather and climate?

Here we argue that this behavior in models *does* appear to be relevant to real-world convection and climate. Certainly, the study of convective self-aggregation is leading to exciting new insights into processes that allow convection to interact with its environment in models. There are encouraging signs that these processes may operate in nature too, as we discuss below. There are also some aspects of self-aggregation in models that conflict with observations, and many aspects that need more observational study.

This paper is organized as follows. In the remainder of this section, we motivate the study of aggregation as a means of understanding real-world climate and review the literature on observations of organized convection and convective aggregation. Section 2 presents a fairly brief review of processes important for self-aggregation and the maintenance of aggregated convection in idealized simulations, with a focus on aspects of these processes that could be targeted in observational studies. We then discuss observational pathways toward assessing the relevance of the idealized framework for real-world applications, including some new results comparing humidity profiles from radiosondes with humidity profiles from idealized self-aggregation, in Sect. 3. Section 4 provides observational perspectives on the possible interaction between convective aggregation and climate change, while Sect. 5 proposes novel approaches to observing convective aggregation, including ideas for new satellite studies and ground-based networks; this is followed by our conclusions.

### Importance of Aggregation

**Fig. 1:**
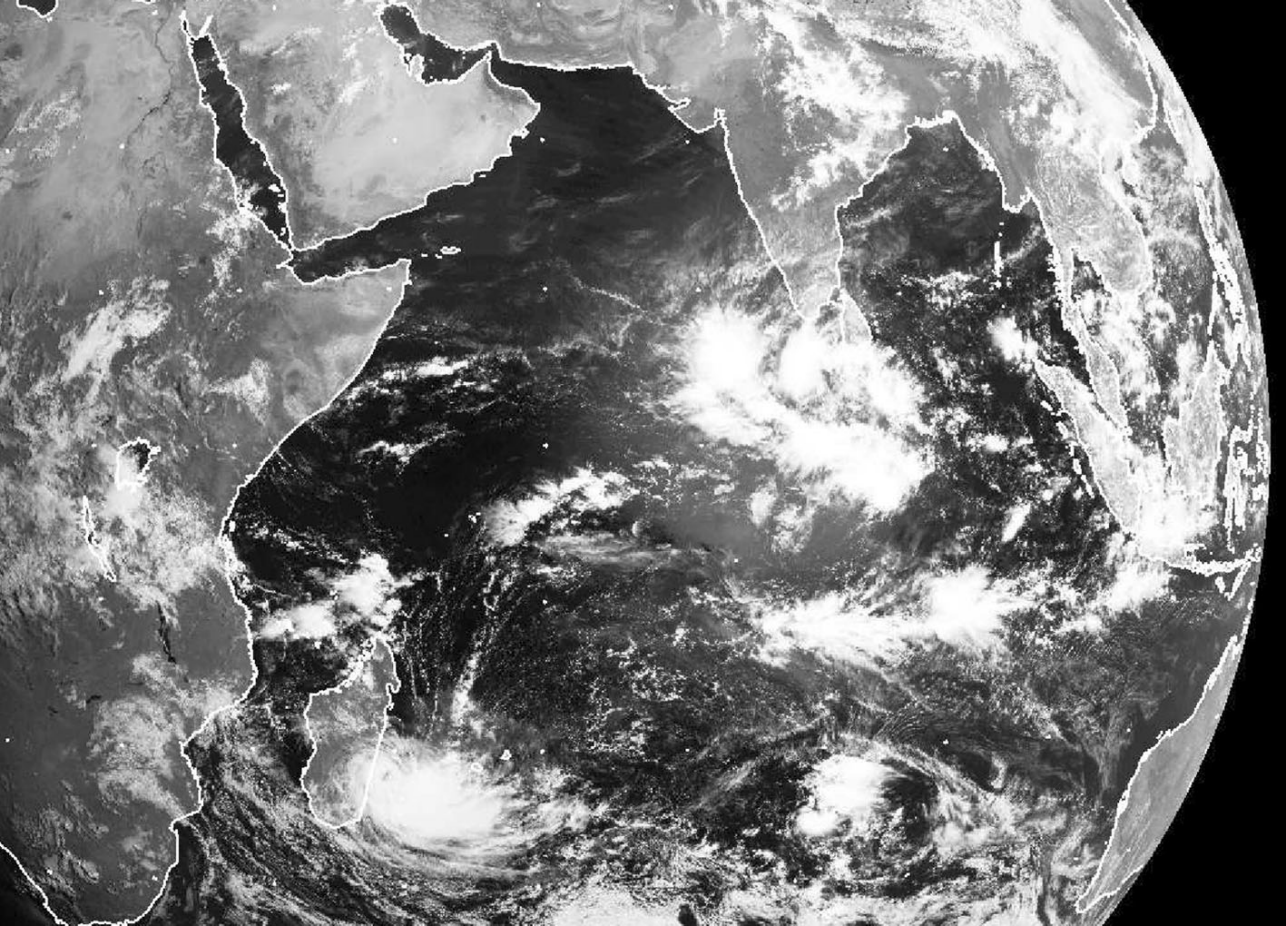
A visible satellite image showing an active Madden–Julian Oscillation (MJO) event on 8 April 2009 and convection organized over a wide range of scales. Image taken from the NERC Satellite Receiving Station, Dundee University, Scotland http://www.sat.dundee.ac.uk/

Convective clouds exhibit a very large diversity of spatial organization, ranging from spatially random distributions to coherent structures such as mesoscale cloud clusters, cloud streets, and squall lines up to cloud envelopes of planetary scale (Fig. 1). For many decades, studies of convective organization have been developed by mesoscale meteorologists and weather forecasters, motivated by the wish to understand why convection would organize in one form rather than another, and by the evidence that the organization of convection matters for the prediction of severe weather. Over the last decade, however, the ability to study the organization of convection with models running at increasingly fine resolution over increasingly large domains has led to new perspectives and to a new line of questioning: Does it make any difference *for climate* whether convection organizes in one form or another?

It has long been recognized that convective organization influences the diabatic heating profile of the atmosphere and thus affects the mean large-scale atmospheric circulation (e.g., Hartmann et al. 1984). More recent numerical studies show that the clumping of convection can occur spontaneously even in the absence of external drivers such as inhomogeneous surface boundary conditions or equatorial wave dynamics (e.g., Held et al. 1993; Bretherton et al. 2005; Muller and Held 2012; Wing and Emanuel 2014) and that this behavior, referred to as convective self-aggregation, may be considered as a fundamental instability of radiative–convective equilibrium (Emanuel et al. 2014). Could tropical phenomena such as tropical cyclones or Madden–Julian Oscillation (MJO) events represent manifestations of this self-aggregation behavior at different spatial scales (Khairoutdinov and Emanuel 2010, 2013; Arnold and Randall 2015)? Answering this question would provide new opportunities to understand and to predict these phenomena through completely novel approaches.

Numerical studies of convective aggregation also show that the clumping of convection is associated with changes in the large-scale state, including a drying of the atmosphere, a shrinking of upper-tropospheric clouds, and an enhanced ability of the atmosphere to lose heat to space (e.g., Wing and Emanuel 2014; Wing and Cronin 2016; Holloway and Woolnough 2016; Bony et al. 2016). Self-aggregation in numerical models also exhibits some temperature dependence (Khairoutdinov and Emanuel 2010; Wing and Emanuel 2014; Emanuel et al. 2014; Coppin and Bony 2015; Wing and Cronin 2016). The combination of these different findings implies that changes in convective organization could occur under climate change, potentially affecting the water vapor and cloud feedbacks. These numerical results shed new light on the role that convective aggregation might play in climate (Mapes 2016): Could a sensitivity of convective aggregation to temperature modulate climate sensitivity and hydrological sensitivity (Khairoutdinov and Emanuel 2010; Mauritsen and Stevens 2015; Bony et al. 2015)? In a warmer climate, could it play a role in the intensification of the MJO (Arnold and Randall 2015; Arnold et al. 2015) or in the narrowing of tropical rain belts (Bony et al. 2016)?

Many of these exciting scientific questions primarily stem from numerical investigations. However, numerous studies (e.g., Stephens et al. 2008; Muller and Bony 2015; Wing and Cronin 2016; Holloway and Woolnough 2016; Silvers et al. 2016; Tompkins and Semie 2017) demonstrate that the behavior of convective aggregation in models can be sensitive to aspects of the experimental setup (such as the size of the domain) and/or to the models themselves (e.g., horizontal resolution, the representation of diabatic processes or the parameterization of subgrid-scale mixing).

To move forward, we must therefore expand our study of the aggregation of convection using observations. We must probe links between processes in idealized self-aggregation and observed convective organization and also confront differences between idealized frameworks and the real world. We first present a review of relevant literature below before addressing these topics in the following sections.

### Literature Review: Observational Studies of Convective Organization

There is a rich history of observational work on convective clustering and organization, much of which details the climatology and life cycles of these systems. The primary source of data for this observational work is infrared and visible images from geostationary satellites, dating back to at least Arkin (1979) and encompassing Velasco and Fritsch (1987), Miller and Fritsch (1990), Laing and Fritsch (1993a, b), Machado and Rossow (1993), Mapes and Houze (1993), Laing and Fritsch (1997), Zuidema (2003), and Hennon et al. (2012), but some more recent studies have also used other types of satellite data such as precipitation radar (Nesbitt et al. 2000; Schumacher and Houze 2003; Futyan and Genio 2007; Peters et al. 2009), microwave measurements of column water vapor (CWV) (Mapes et al. 2009), and scatterometer winds (Mapes et al. 2009). While cloud clusters are often identified by searching for large, contiguous cold cloud shields, more advanced techniques search for the combined signature of deep convection and extensive stratiform cloud and precipitation area. For example, higher stratiform rain fractions are associated with organized convection, which can be diagnosed from satellite precipitation radar data (Schumacher and Houze 2003), as are large optical thicknesses and low-cloud top pressures, which can distinguish a particular cloud regime (Tselioudis et al. 2010; Tan et al. 2013).

A significant fraction of the observational work on organized convection has focused on mesoscale convective systems (MCSs), or a subset of them known as mesoscale convective complexes (MCCs), which occur in both the tropics and mid-latitudes. A global climatology of MCCs, which are identified by a large (>10$^5$ km$^2$), long-lasting (>6 h), quasi-circular cold cloud shield, was compiled by Laing and Fritsch (1997) based on previous regional studies (Miller and Fritsch 1990; Laing and Fritsch 1993a, b; Velasco and Fritsch 1987).

Other studies have detailed the properties of, more generally, tropical cloud clusters and deep convective systems. This includes studies on the structural characteristics and radiative properties of tropical high cloud systems (Machado and Rossow 1993), the life cycles of deep convective systems (Futyan and Genio 2007; Mapes et al. 2009), the size distribution of cloud clusters (Mapes and Houze 1993; Roca and Ramanathan 2000; Zuidema 2003; Peters et al. 2009), and the spatial and temporal variability in cloud clusters and their efficiency at producing tropical cyclones (Hennon et al. 2012). Studies have also pointed out significant self-similarity between MCSs and convectively coupled equatorial waves (Mapes et al. 2006; Kiladis et al. 2009).

Despite the fact that the occurrence of mesoscale organized convection makes up a small fraction of the total frequency of cloud/precipitation features in the tropics (<6%, Mapes and Houze 1993; Nesbitt et al. 2000; Tan et al. 2013), it contributes a significant proportion of total tropical cloudiness^1^ and about half of total tropical precipitation.^2^ Tropical cloud clusters therefore may modulate the radiative heating of the surface and atmosphere (e.g., Machado and Rossow 1993) and strongly influence the large-scale circulation, moisture distribution, and hydrological cycle. There is observational evidence that the frequency of organized convection has increased across the tropics over the past $\sim$30 years (Tselioudis et al. 2010) and that most of the regional increases in tropical precipitation over that period are associated with this increase (Tan et al. 2015). In addition to their contribution to tropical cloudiness and precipitation, tropical cloud clusters also play an important role as precursors to tropical cyclones, with globally 6.4% of tropical cloud clusters developing into tropical cyclones each year (Hennon et al. 2012).

Another observational finding which may be relevant to self-aggregation is the evidence that tropical precipitation has properties like those of a critical phenomenon. Peters and Neelin (2006) found that there is a power law increase in precipitation with CWV above a critical CWV value, and a sharp peak in the variance of precipitation at the critical value. Holloway and Neelin (2009) further found that free-tropospheric moisture plays a key role in the transition to deep convection and linked the increase in precipitation with CWV to an increase in the buoyancy of entraining plumes, which relates to the proposed moisture–convection feedback in self-aggregation. Neelin et al. (2009) also noted that the atmosphere is near criticality a larger fraction of the time when it is over warm sea surface temperatures (SSTs). Peters et al. (2009) found that precipitation clusters exhibited scale-free size distributions including much larger clusters near-critical CWV than below it, suggesting a possible link between clustering within the moist convective regions in idealized self-aggregation and near-critical CWV values.

While the literature on observations of tropical cloud clusters is extensive (only a small segment of which was reviewed here), only a few studies have specifically looked for processes related to modeled self-aggregation using observations. The first such paper, Tobin et al. (2012), used geostationary satellite infrared brightness temperature in snapshots of large tropical latitude-longitude boxes ($10^{\circ }\times 10^{\circ }$) to categorize observations by their degree of convective organization. To do this, they devised the Simple Convective Aggregation Index (SCAI) as a combined measure of cluster number and inter-cluster distance, with cluster pixels defined as having brightness temperature below 240 K and with larger SCAI corresponding to a less aggregated state. They found that cluster number was statistically sufficient to discriminate between different levels of aggregation, so they often used the number of clusters as a metric for the degree of aggregation, with fewer clusters corresponding to a more aggregated state. By controlling for measures of box-mean convective intensity and large-scale forcing, including rainfall from microwave satellite data, SST from infrared satellite data, and vertical velocity from reanalyses, they could compare atmospheric conditions for varying amounts of convective organization in a way that was analogous to comparing different stages of aggregation in idealized models.

**Fig. 2:**
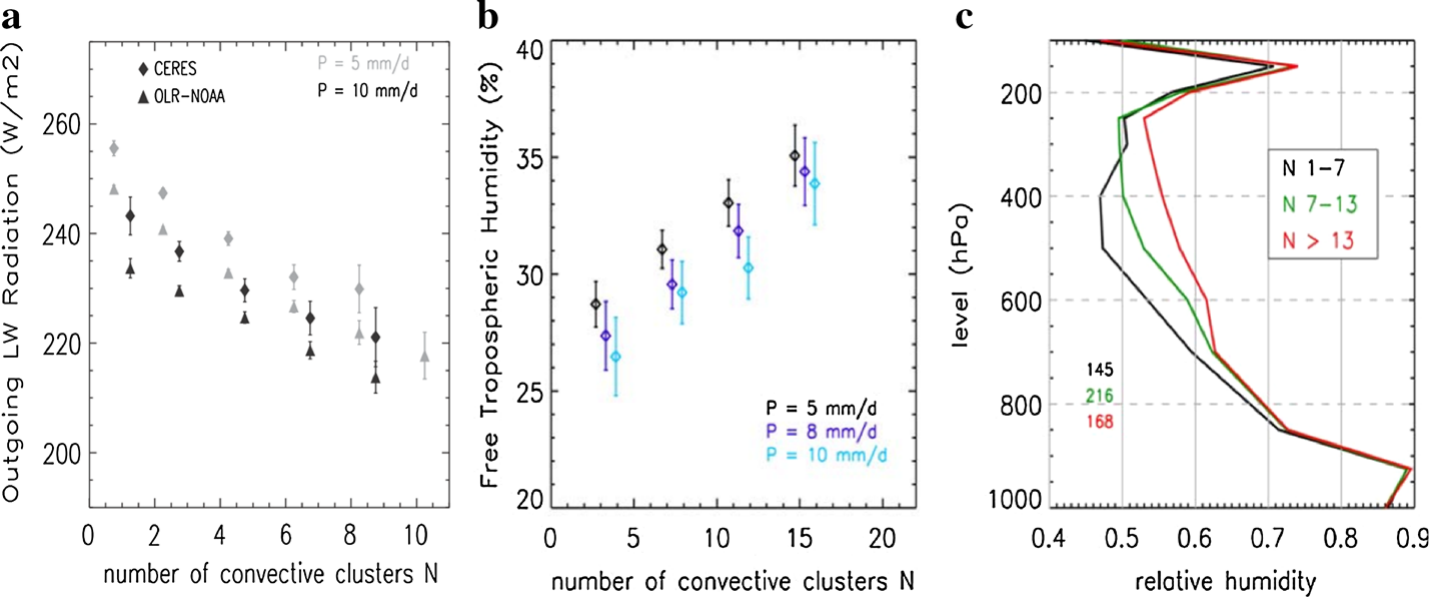
**a** Composites over many $10^{\circ }\times 10^{\circ }$ snapshots of domain-averaged OLR from CERES and NOAA for two different average rain rates for different satellite-derived cluster numbers, with fewer clusters representing more aggregated convection. **b** Similar analysis for $3^{\circ }\times 3^{\circ }$ snapshots of domain-averaged free-tropospheric humidity derived from Meteosat Tb in the WV channel, for three different average rain rates. **c** Domain-averaged AIRS relative humidity composited on the same $3^{\circ }\times 3^{\circ }$ snapshots as in **b** for three cluster number bins for a precipitation rate of $8\,\hbox {mm}\,\hbox {day}^{-1}$. Figures from Tobin et al. (2012) (panel a) and Tobin et al. (2013) (panels b, c)

Tobin et al. (2012) found several similarities between their observational analyses and idealized simulations of self-aggregation. Holding large-scale SST and rainfall constant, they found that more aggregated states had a drier free troposphere in the non-convective environment and, consequently, in the domain as a whole. They also found an increase in outgoing longwave radiation (OLR) at the top of the atmosphere (by as much as $30\,\hbox {W}\,\hbox {m}^{-2}$) with aggregation (Fig. 2a), mainly because of a reduction of mid-level and upper level cloudiness. These main conclusions, in agreement with all studies of idealized self-aggregation in models, were also supported by a related paper, Tobin et al. (2013), which looked at smaller ($3^{\circ }\times 3^{\circ }$) domains using higher-resolution satellite brightness temperature data (Fig. 2b, c), as well as Stein et al. (2017), which analyzed the vertical cloud structure for different SCAI values using CloudSat–CALIPSO data.

On the other hand, Tobin et al. (2012, 2013) found some results that were inconclusive, mixed, or contradictory when compared with modeling studies. For instance, Tobin et al. (2012) found that surface turbulent heat fluxes increased both inside and outside convective regions when aggregation increased, whereas Tobin et al. (2013) found little sensitivity of these fluxes to aggregation at the smaller scales they investigated (although this discrepancy could be due to limitations in satellite retrievals of surface fluxes). In idealized simulations, surface fluxes generally increase with self-aggregation (e.g., Bretherton et al. 2005; Wing 2014; Holloway and Woolnough 2016), with the increase due to larger wind speeds in general and larger air–sea enthalpy disequilibrium in the dry environment (Wing and Emanuel 2014). (Note that this modest increase in surface fluxes for idealized models is also consistent with slightly larger atmospheric radiative cooling rates and precipitation rates in radiative–convective equilibrium after aggregation has occurred.) Tobin et al. (2012, 2013) also found that the top-of-atmosphere net radiation budget was not significantly affected by aggregation because increased OLR was offset by decreased reflected shortwave radiation. This differs from idealized simulations discussed by Wing and Cronin (2016), in which an increase in low-cloud fraction with aggregation left reflected shortwave largely unchanged, leading to a net loss of radiation at the top of atmosphere for aggregated conditions. Tobin et al. (2013) and Stein et al. (2017) both found evidence for an increase in low-cloud fraction with aggregation, while Tobin et al. (2012) found the opposite, so the models are supported by at least some observational studies regarding low-cloud changes.

In the next section, we briefly review processes found to be important for self-aggregation in models with a focus on links to observed convective organization.

## Observational Perspectives on Processes Important for Idealized Convective Aggregation

There are longstanding attempts to reconcile the well-observed clumping of tropical convection with simple theory (e.g., Mapes 1993). Randall and Huffman (1980) proposed that clumping occurs when clouds can create an area around themselves that is more favorable for future convection than areas further away. Numerical studies of self-aggregation have identified multiple processes involving convection–moisture–radiation feedbacks that are capable of doing exactly that. The diversity of processes that can lead to convective aggregation may explain why it has been observed by multiple different modeling groups using very different models, from high-resolution cloud-resolving models to global climate models (GCMs) with parameterized convection. Additionally, different feedbacks that lead to aggregation may be excited by different initial conditions.

We will mostly discuss self-aggregation in idealized settings: radiative convective equilibrium (RCE) over constant uniform SST in non-rotating, three-dimensional, doubly periodic square domains, though some rectangular and aquaplanet simulations will occasionally be discussed as well. It is worth noting that self-aggregation has been shown to be robust to the presence of rotation (Bretherton et al. 2005; Khairoutdinov and Emanuel 2013; Bretherton and Khairoutdinov 2015; Davis 2015; Wing et al. 2016), vertical shear (Bretherton et al. 2005), diurnal cycle (Wing and Cronin 2016), two-dimensional or three-dimensional settings (Held et al. 1993; Jeevanjee and Romps 2013), and an interactive ocean mixed layer (Bretherton et al. 2005; Hohenegger and Stevens 2016), and to occur as well in global climate simulations with parameterized convection in aquaplanet non-rotating settings (Coppin and Bony 2015; Popke et al. 2013; Reed et al. 2015).

In this section, we briefly review the various processes leading to the self-aggregation of convection in RCE simulations and the metrics used to quantify them, including the physical processes that lead to aggregation from homogeneous initial conditions as well as those which can maintain convective aggregation once it is established. We focus on those which could be targeted in observations; a more complete review can be found in Wing et al. (2017).

### Metrics to Quantify Feedbacks

Several methods have been proposed to analyze the leading order feedbacks in simulations (and also possibly in observations). They all share the methodology of stratifying the data by vertically integrated moist static energy (MSE). In the tropics, weak temperature gradients imply that horizontal variability of MSE is largely dictated by variability in CWV. Using this methodology, different variables can be moisture-ranked.

Wing and Emanuel (2014) introduced an analysis framework employing a budget for the spatial variance of MSE. Self-aggregation is associated with a very strong increase in MSE variance. The equation for the time evolution of MSE variance allows one to estimate the various contributions to the enhanced MSE variability. The terms of this budget include the horizontal convergence or divergence of MSE, as well as the direct diabatic contributions from radiative and surface fluxes, i.e., whether a heating/moistening diabatic tendency reinforces (positive feedback) or smoothes (negative feedback) MSE gradients. The potential use of observations to calculate the diabatic terms in the MSE spatial variance budget is discussed more in Sect. 2.4.

Note that these diabatic terms include the direct diabatic effects of radiative and surface flux feedbacks, not the circulation that the diabatic terms generate. For instance a positive shortwave (SW) feedback means anomalous SW heating in the high-MSE region and/or anomalous SW cooling in the low-MSE region, thereby enhancing the MSE gradient. The diabatic feedback term does not account for the dynamical response to this SW heating distribution, which can also transport MSE up- or down-gradient. This transport is a component of the horizontal convergence term, but is not explicitly diagnosed separately from the other dynamical contributions in this framework. Another related issue is that these diagnostics are based on vertical integrals and hence do not explicitly capture the sensitivity to the vertical distribution of diabatic forcings found in Muller and Bony (2015). Indeed diabatic tendencies applied at different heights can yield different MSE transports since MSE varies strongly with height.

An assessment of both the direct diabatic effect and the indirect circulation and MSE transport corresponding to a heating anomaly is achieved in model simulations with sensitivity runs in which diabatic terms are horizontally homogenized, removing both direct and indirect effects, as done in Muller and Held (2012). This is obviously not possible with observations. The remaining option is to analyze the circulation generated by diabatic forcing and infer the MSE transport as done in Holloway and Woolnough (2016). The visualization of the MSE transport is usually done with a stream function in moisture and height space (Bretherton et al. 2005; Muller and Held 2012; Holloway and Woolnough 2016). This quantifies the energy transport between the dry region and the moist region and hence determines whether it is up-gradient, which is typical of aggregation in idealized model studies (e.g., Bretherton et al. 2005; Muller and Held 2012) though the total vertically integrated transport is not always up-gradient (e.g., Coppin and Bony 2015). This visualization method is useful in simulations, where vertical profiles of vertical velocity as a function of MSE are available, but it is not clear whether it is applicable to observations. Also, quantifying the role played by radiation in the circulation requires vertical profiles of radiation as a function of MSE (cf. Muller and Bony 2015), which are only beginning to become available in observations (e.g., Haynes et al. 2013). Section 5.3 explores possible approaches to observing these profiles using ground-based instruments.

Bretherton et al. (2005) showed that they could capture the aggregation instability in a semiempirical toy model accounting for the sensitivity of radiative and surface fluxes, as well as MSE convergence, to humidity. In their theoretical paper of convective aggregation, Craig and Mack (2013) take a somewhat similar approach, although the physical processes are modeled differently (in particular the MSE convergence is modeled as a diffusive process). The end result is an expression of the rate of change of humidity as a function of humidity itself $\partial I/ \partial t=f(I)= -\delta V/ \delta I + T$, where *I* is the order parameter (in this case column-integrated free-tropospheric water vapor), *V*(*I*) is a potential function, and *T* is a diffusive transport term. The minima of the functional *V*(*I*) are equilibrium values of humidity. The structure of the functional *V*(*I*) therefore highlights the appearance of multiple equilibria typical of self-aggregation, with the two minima corresponding to the moist and dry solutions. Although this framework allows for the identification of aggregation, it is unclear if it can be used to identify feedbacks involved in the aggregation process. Aggregation from different feedbacks may have different signatures in the functional dependence *V*(*I*).

More work using theory, as well as idealized (and perhaps more realistic) simulations, is desirable to compare conceptual frameworks and metrics of aggregation and determine how these could be applied to observations.

### Initiation Processes

**Fig. 3:**
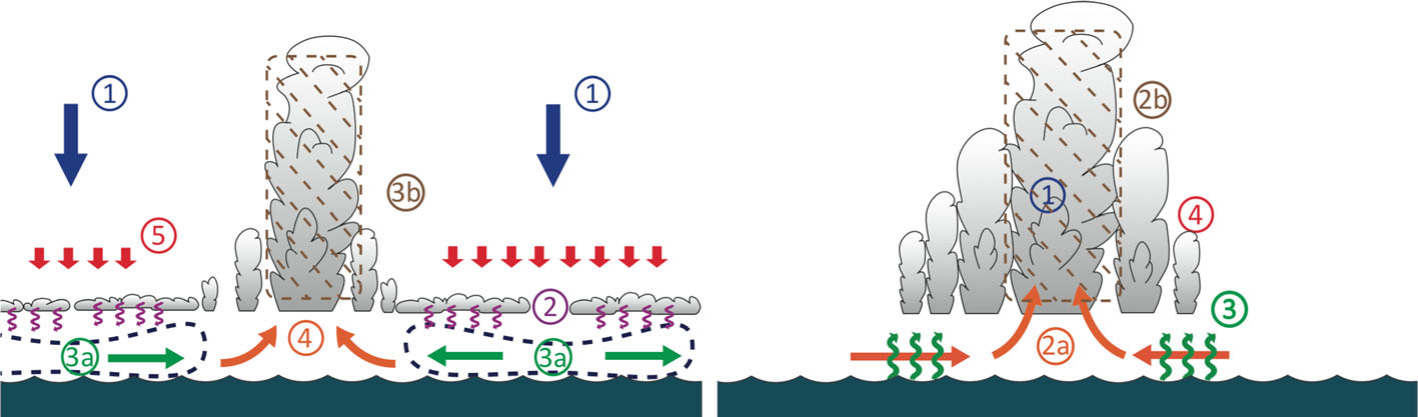
Aggregation processes for: (*left*) cold SSTs with radiatively driven cold pools, and (*right*) warm SSTs with surface flux feedbacks. Figure adapted from Coppin and Bony (2015)

At SSTs close to our current tropical climate (300 K or so), the leading physical process behind the spontaneous self-aggregation of convection seems to be a “radiatively driven cold pool” outside deep convection, as seen in a schematic from Coppin and Bony (2015) (Fig. 3). One or several dry regions appear and expand, with strong longwave radiative cooling and subsidence yielding further drying. Moisture and convection are confined to the rest of the domain, as the dry convection-free region expands. In the following, we briefly review the various physical processes contributing to the formation of this radiatively driven cold pool for temperatures close to current tropical atmospheric temperatures, and then we discuss the sensitivity of these processes to SST. A more complete review can be found in Wing et al. (2017).

#### Longwave Radiation

As mentioned above, aggregation generally begins with the formation of a dry region with strong radiative cooling. The strong longwave cooling in the dry region is largely induced by low-level clouds (Muller and Held 2012; Muller and Bony 2015; Coppin and Bony 2015; Holloway and Woolnough 2016), although clear-sky cooling also contributes (Wing and Emanuel 2014; Wing and Cronin 2016).

Strong subsidence in dry regions, theoretically predicted by the RCE instability study of Emanuel et al. (2014), further promotes the formation of low-level clouds. These in turn enhance the radiative cooling, forming radiatively driven cold pools in dry regions responsible for the clumping of convection in the rest of the domain. Note that these radiatively driven cold pools are colocated with the dry regions and therefore do not mix boundary layer air between moist and dry regions, whereas “conventional cold pools” (defined here as cold pools resulting from downdrafts caused by rain evaporation and/or condensate loading) can propagate from moist to dry regions and tend to slow or weaken aggregation (cf. Jeevanjee and Romps 2013).

#### Surface Fluxes

Feedbacks involving surface enthalpy fluxes favor the initiation of self-aggregation due to larger surface winds in the moist, convecting area, which enhance the up-gradient MSE transport associated with the radiatively driven cold pool discussed above. However, while sensitivity runs with homogenized surface fluxes (no feedback) sometimes do not aggregate (Tompkins and Craig 1998; Bretherton et al. 2005; Wing 2014), they *can* aggregate depending on the domain size, strength of the surface fluxes imposed, and availability of radiative feedbacks (Muller and Held 2012; Holloway and Woolnough 2016). Therefore, surface fluxes feedbacks are not critical for aggregation to occur, at least at current temperatures.

#### Shortwave Radiation

The direct, diabatic effect of shortwave radiation is a positive feedback on aggregation due to variations in the absorption of shortwave radiation by water vapor (Wing and Emanuel 2014), but it is weaker than the longwave and surface flux feedbacks. In sensitivity experiments that include both direct and indirect (dynamic response to the diabatic forcing) effects, shortwave feedbacks slightly oppose aggregation. Either way, the impact of shortwave radiation appears to be secondary, at least at current temperatures.

#### Moisture–Convection Feedbacks

Moisture–convection feedbacks, in which convection moistens the atmosphere and is also more likely to occur in moister conditions, amplify the instabilities leading to self-aggregation (Tompkins 2001; Mapes and Neale 2011; Emanuel et al. 2014). When radiation feedbacks (which are normally required for self-aggregation) are suppressed while rain evaporation is also suppressed (preventing conventional cold pools which can otherwise weaken aggregation), these moisture–convection feedbacks are strong enough to cause aggregation on their own (Muller and Bony 2015; Holloway and Woolnough 2016). This appears to occur through a process similar to the coarsening process in Craig and Mack (2013) in which initial perturbations of a bistable system grow over time. These feedbacks are difficult to quantify directly, even in models, and they would also be difficult to target in observations. A place to start (perhaps using field campaign data) would be to correlate convective activity with moist (high MSE) locations and then to estimate the transport of MSE (part of the convergence term in the MSE spatial variance budget) due to circulations forced by this anomalous convective heating.

### Sensitivity to SST

Several aspects of self-aggregation are sensitive to SST, such as its initiation mechanisms, spatial scale, and perhaps degree of organization (Khairoutdinov and Emanuel 2010; Wing and Emanuel 2014; Emanuel et al. 2014; Wing and Cronin 2016; Coppin and Bony 2015; Abbot 2014; Holloway and Woolnough 2016). It is worth noting that self-aggregation is found at temperatures much colder than our current climate, including 243 K in snowball Earth simulations (Abbot 2014) and 280 K in long-channel experiments (Wing and Cronin 2016), as well as much warmer (e.g., 310 K, Wing and Cronin 2016). The radiatively driven cold pools discussed above seem to be most efficient at cold and current temperatures (Fig. 1, Coppin and Bony 2015), possibly because climate models with strong positive low-cloud feedback (like the model used in that study) do not have any low clouds at high temperatures. However, cloud radiative feedbacks may behave differently at much colder temperatures (Wing and Cronin 2016) and clear-sky longwave feedbacks are favored by warm temperatures (Emanuel et al. 2014). At warm temperatures, surface-flux-wind feedbacks in the high-MSE convective region are the leading mechanism for self-aggregation in GCM simulations (Fig. 1, Coppin and Bony 2015).

In their semiempirical model of self-aggregation based on cloud-permitting simulations at present-day temperatures, Bretherton et al. (2005) found a slightly stronger sensitivity of radiative fluxes to moisture than that of surface fluxes. These sensitivities are likely to be different at different temperatures.

### Maintenance Processes

Given that the real tropical atmosphere is never starting from a homogeneous background state, as in the idealized simulations, the processes that maintain existing convective aggregation may be easier to observe than those initiating it. While the strongest positive feedbacks in the early stages of idealized self-aggregation are usually found in the dry region, at later times strong consistently positive feedbacks are found only in the moist region. Muller and Held (2012) and Muller and Bony (2015), which find low clouds to be necessary for the initiation of aggregation using mechanism denial experiments, find that low clouds are not necessary to maintain self-aggregation in their simulations. Instead, high clouds in the moist regions and clear-sky longwave feedbacks can maintain aggregation (Muller and Held 2012; Wing and Emanuel 2014; Muller and Bony 2015; Wing and Cronin 2016). Possible sensitivity of these maintenance processes to SST is discussed in Sect. 4 below.

Surface flux feedbacks are neither necessary nor sufficient to maintain non-rotating aggregation (Holloway and Woolnough 2016), at least at current climate temperatures. Indeed, the surface flux feedback becomes negative in later stages of non-rotating aggregation, due to the opposing influences of surface winds and air–sea enthalpy disequilibrium (Wing and Emanuel 2014). However, surface flux feedbacks could behave differently in simulations with an interactive SST calculated from surface energy balance.

Quantifying the strength of these maintenance feedbacks in observations would be desirable. As in the simulations discussed earlier, the radiative and surface flux feedbacks could be diagnosed by their contributions to the MSE spatial variance budget. These require simultaneous measurements over a large area of the top-of-atmosphere and surface radiative fluxes, as well as observed surface enthalpy fluxes and vertically integrated MSE. Alternatively, an MSE temporal variance budget could be computed at a given location, assuming that with time, both the dry and moist regions of aggregated convection would pass over the station. Methods for estimating quantities needed to calculate these terms using satellite data are explored in Sect. 5.1. The strength of the radiative and surface flux feedbacks could also be correlated with the degree of aggregation as measured by SCAI (defined in Tobin et al. 2012). Tobin et al. (2013) used SCAI calculated from observations to suggest that intraseasonal variations of aggregation tend to amplify dynamical anomalies. Similarly, recent work compositing on MJO events during the DYNAMO field campaign has shown that radiation and, to a lesser extent, surface heat fluxes play an important role in amplifying MJO variability (Sobel et al. 2014), revealing potential links to the aggregation work proposed here.

## Comparing the Idealized World to the Natural World

In addition to process-oriented studies, observations can also be used to test the realism of the mean state, variability, and convective characteristics of the idealized models. Here we explore similarities and differences between these aspects of idealized simulations of self-aggregation and observations. We also discuss processes that are not usually captured by idealized models, such as ocean interaction. Linking self-aggregation processes to environments with further complexity, such as non-uniform SST or the effects of land and orography, is not addressed here but deserves future investigation. The motivation for this section is that, in order to have confidence in the relevance of self-aggregation processes found in idealized simulations for observed convective organization, we need to be able to understand and explain differences between the idealized world and the natural world.

### Time Scales of Self-Aggregation

One common critique of idealized self-aggregation is that the time scale of the aggregation process is much longer than typical time scales for observed convective organization. This is a valid concern, but there are several rebuttals which are discussed below. First, there is a broad range of time scales for self-aggregation and disaggregation in the literature, and these appear to depend on model, domain size, resolution, initial conditions, SST, and the inclusion or suppression of processes such as conventional cold pools. Second, self-aggregation from homogeneous initial conditions includes the spin-up of small-scale convective activity and clustering without pre-existing large-scale features, and while we can learn a lot from these early stages they are not likely to occur simultaneously across a large region in the real world where asymmetries are always present. Third, while it is likely that the processes important for idealized self-aggregation are not important for all types of convective organization in nature, and may be less important for rapidly organizing convective systems, some types of organized convection (particularly on longer time scales) do show intriguing links to self-aggregation.

As mentioned in Wing et al. (2017), self-aggregation in idealized models can take 15–100 days or more to reach a relatively stable aggregated state when starting from homogeneous initial conditions (though the longer time scales likely relate to an initial gestation period in some simulations which occurs before aggregation has started at all). There is some sensitivity of this to domain size and grid scale (Muller and Held 2012). When rain evaporation and conventional cold pools were suppressed, Holloway and Woolnough (2016) found that the time scale decreased to only 8 days as opposed to 16 days in their control run, supporting the idea proposed in Jeevanjee and Romps (2013) that conventional cold pools slow or suppress aggregation in idealized simulations. Perhaps also relevant to understanding processes that keep convective clusters organized in nature, Muller and Held (2012) and Holloway and Woolnough (2016) both found a disaggregation time scale (which is the time needed to return to a less aggregated equilibrium) as small as 10 days when simulations were initialized with an aggregated state and then interactive radiation was suppressed.

Wing (2014) found that the spatial MSE variance grew with an e-folding time of $\approx$11–13 days. As mentioned in Wing et al. (2017), this kind of exponential growth will lead to much larger scales in a given amount of time when starting from larger initial clustering, as is typically found in nature. In other words, much of the time scale for self-aggregation from homogeneous initial conditions may not be especially relevant to comparisons with nature because these time periods involve spinning up mesoscale activity from extremely small initial length scales (and may also involve gestation periods before aggregation begins at all). In fact, convective cluster growth across scales (but especially at larger scales) was found to be linked to radiative feedbacks in near-global RCE channel runs (including rotation) in Bretherton and Khairoutdinov (2015), with e-folding time scales of 6–14 days. Those authors suggest that diabatic feedbacks (mainly longwave radiation feedback) may be especially important for large-scale convective organization such as the MJO.

### Mean Wind and Wind Shear

Most idealized RCE studies have no imposed mean wind or wind shear. While wind shear can act to enhance some kinds of mesoscale organization such as squall lines (e.g., Houze 2004; Muller 2013), it has also been shown to slow or prevent self-aggregation in idealized simulations such as those in Bretherton et al. (2005) and Khairoutdinov and Emanuel (2010), although the latter found that there was hysteresis, since an already aggregated state did not disaggregate with some levels of imposed shear.

Nonzero mean vertical velocity due to large-scale circulations is common in regions containing organized tropical systems in nature but cannot occur for the domain mean in a typical RCE setup. Global-scale simulations, however, do represent these circulations (e.g., Coppin and Bony 2015), and smaller RCE simulations can impose them (e.g., Su et al. 2000) or parameterize them using reference profiles and assumptions of weak temperature or pressure gradients (e.g., Sessions et al. 2016). As these kinds of modeling studies progress, there will be more opportunities to evaluate their simulated relationships between aggregation and large-scale circulations using observations.

### Humidity Profiles

**Fig. 4:**
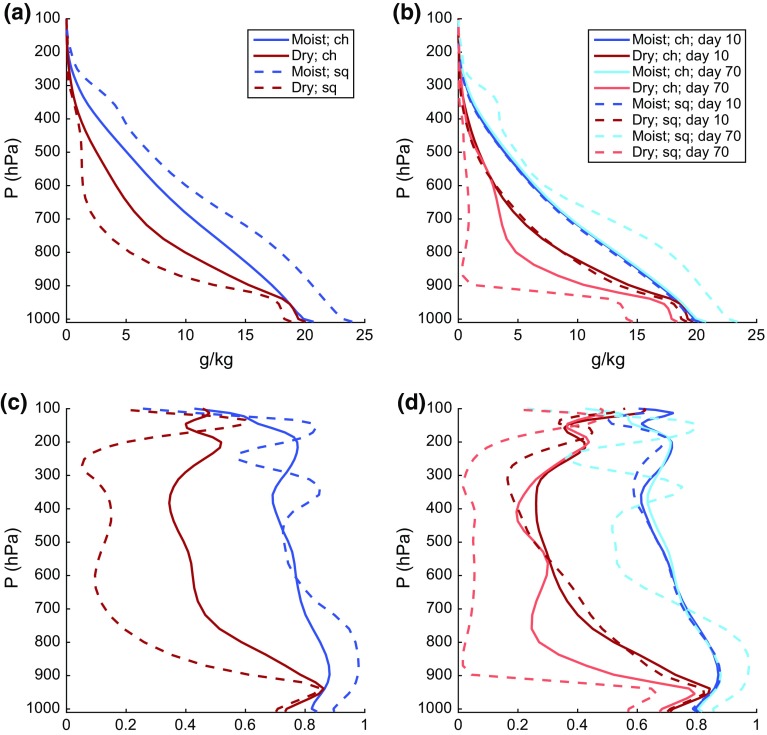
Profiles of water vapor mixing ratio (*top panels*) and relative humidity (*bottom panels*) in simulations in elongated channel (*solid lines*) and square (*dashed lines*) domains. The *left panels* define moist regions (*blue*) as area where $\text {CWV} \ge 0.8 \,\, \text {max(CWV)}$ [or $\text {CRH} \ge 0.8 \,\, \text {max(CRH)}$ in panel **c**], dry regions (*red*) defined as the rest of the domain; the profiles are averaged over the last 10 days of the simulation. The *right panels* show profiles from the moistest (*shades of blue*) and driest (*shades of red*) 10% of the domain, according to CWV [or CRH in **d**]. Profiles from both the developing (5-day average centered at day 10; *lighter colors*) and mature (5-day average centered at day 70; *darker colors*) stages of aggregation are plotted. **a** *q* where CWV >/< 0.8 max (CWV), **b** *q* in moistest/driest 10% of domain, **c** RH where CRH >/< 0.8 max (CRH) and **d** RH where CRH >/< 0.8 max (CRH)

One of the potentially unrealistic aspects of self-aggregation as seen in idealized models that needs to be reconciled with observations is the presence of very dry humidity profiles that occur in the non-convecting areas of the domain. Since humidity plays a key role in all of the feedbacks important for self-aggregation, it is especially important to investigate this issue. To that end, we include here some examples of humidity profiles (and the related radiative heating profiles) in moist and dry areas of simulated self-aggregation. These profiles are from the simulations presented by Wing and Cronin (2016). We show profiles from two simulations: one with a square domain that is 1536 km $\times$ 1536 km in the horizontal (*sq*) and one that is an elongated channel with dimensions of 12,288 km $\times$ 192 km in the horizontal (*ch*). The *sq* simulation has one circular, intensely precipitating moist cluster while the *ch* simulation has multiple moist and dry bands. All other aspects of the simulations are identical.^3^ Fig. 4 shows water vapor mixing ratio and relative humidity in the moist and dry areas, averaged over the last 10 days of the two simulations. In Fig. 4a, the “moist” area is defined as the area where the CWV is greater than 80% of the maximum CWV found in the last 10 days of simulation. The rest of the domain is classified as the “dry” area. Profiles using an alternate definition of moist and dry areas are shown in Fig. 4b, in which the “dry” and “moist” areas are the driest 10% and moistest 10% of the domain according to CWV. Here, we show profiles from both the developing and mature stage of aggregation, using 5-day averages centered at day 10 and day 70, respectively. Figure 4c, d show similar plots to Fig. 4a, b but for relative humidity, with ranking done according to column relative humidity (CRH, defined as CWV divided by column-integrated saturation specific humidity) instead of CWV.

**Fig. 5:**
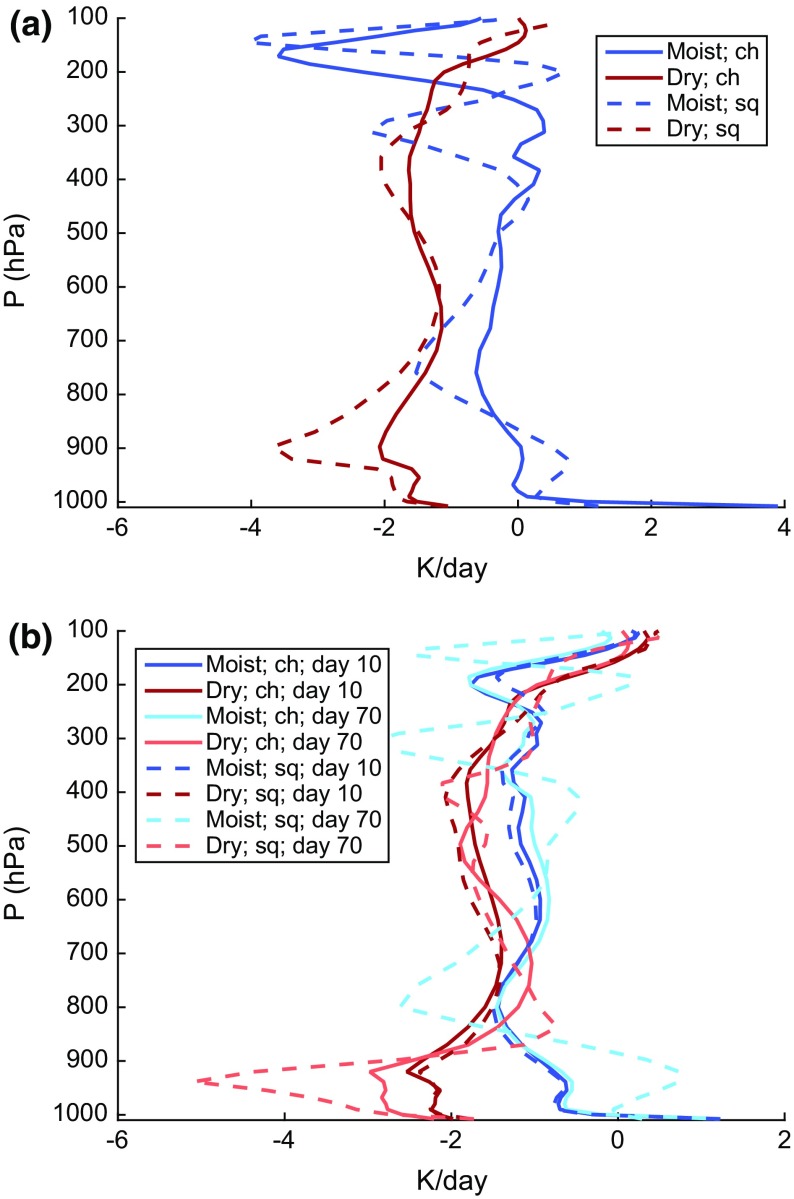
Profiles of radiative heating rate in simulations in elongated channel (*solid lines*) and square (*dashed lines*) domains. **a** Moist regions (*blue*) as area where $\text {CWV} \ge 0.8 \,\, \text {max(CWV)}$, dry regions (*red*) defined as the rest of the domain; the profiles are averaged over the last 10 days of the simulation. **b** Profiles from the moistest (*shades of blue*) and driest (*shades of red*) 10% of the domain, according to CWV. Profiles from both the developing (5-day average centered at day 10; *lighter colors*) and mature (5-day average centered at day 70; *darker colors*) stages of aggregation are plotted. **a** *Q*_rad_ where CWV >/< 0.8 max (CWV) and **b** *Q*_rad_ in moistest/driest 10% of domain

As shown in Fig. 4, the water vapor mixing ratio and relative humidity are substantially reduced in the dry regions relative to the moist regions at all levels (including the boundary layer), but most strongly in the mid-troposphere. The difference between the dry and moist regions is stronger for the *sq* simulation than the *ch* simulation, reflecting the more extreme (and arguably less realistic) aggregation that occurs in square domain simulations. There is significantly more radiative cooling in the dry regions than the moist regions, especially in the lower troposphere (Fig. 5), which further amplifies the anomalies.

These results naturally lead to several questions about how representative these idealized simulations are of humidity variability in the real tropics. The behavior of the humidity profiles across the different evolutionary states of aggregation in the simulations (from developing to mature aggregation) is interesting; substantial drying is present in the upper troposphere as early as day 10, but drying of the middle–lower troposphere and boundary layer does not appear until later in the simulation (Fig. 4b, d). One potential avenue of research to link this to observations is to relate the evolution of humidity in the dry regions and the stage of aggregation to the altitude depth of the bimodality of water vapor (Mapes 2001, 2016; Zhang et al. 2003). However, a more basic starting point is to determine whether humidity in the tropics exhibits a similar range of variability between dry and moist conditions: do humidity profiles as dry as the ones in simulated aggregation exist in the real tropics?

**Fig. 6:**
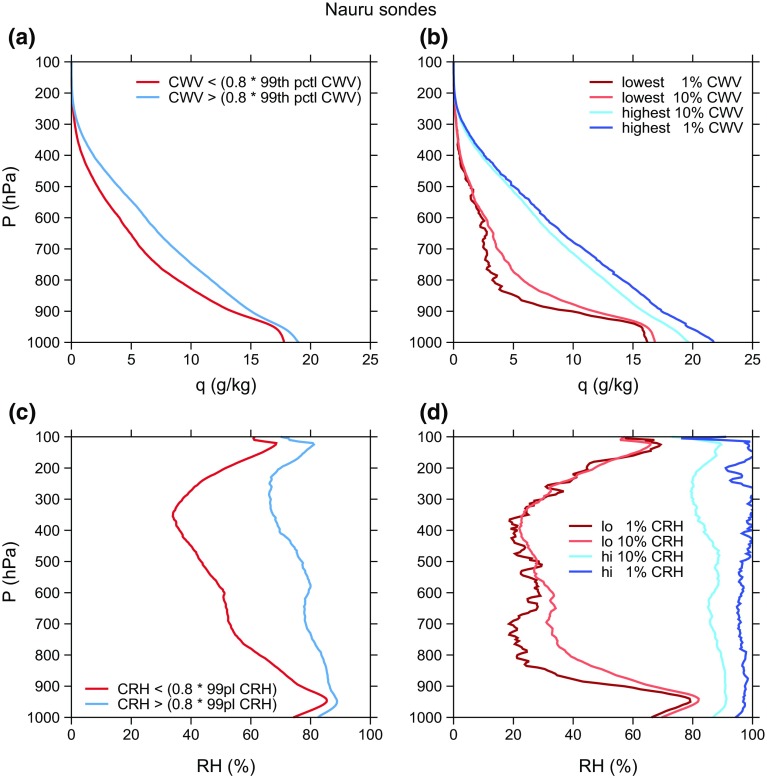
Nauru radiosondes: **a** mean profiles of water vapor specific humidity (g kg$^{-1}$) for all sondes with CWV greater than 0.8 times the 99th percentile of CWV and for all sondes less than this threshold, **b** mean profiles for all sondes in the lowest 1%, lowest 10%, highest 10%, and highest 1% as ranked by CWV, **c** mean profiles of relative humidity (%) for all sondes with CRH greater than 0.8 times the 99th percentile of CRH and for all sondes less than this threshold, and **d** mean profiles of relative humidity for all sondes in the lowest 1%, lowest 10%, highest 10%, and highest 1% as ranked by CRH

As a first step toward answering this question, we compare the humidity data from the idealized simulations in Wing and Cronin (2016) to twice-daily radiosondes from Nauru in the Pacific warm pool. Figure 6 shows humidity profiles from 5 years of the Nauru radiosondes. These data span the period from April 1, 2001 to August 16, 2006 and are from the former Atmospheric Radiation Measurement (ARM) site (Mather et al. 1998; Long et al. 2016). The data, which include 3491 retained sondes, are described in more detail in Holloway and Neelin (2009). Figure 6a shows mean specific humidity profiles of two subsets of sondes divided by a CWV threshold of 0.8 times the 99th percentile of CWV (55  mm, the 63rd percentile). Despite coming from a range of SSTs which are generally a few degrees cooler than 305 K, these profiles look quite similar to the mixing ratio profiles from the *ch* simulation shown in Fig. 4a, while the *sq* simulation in that figure shows much more spread between moist and dry profiles. Similarly, the extreme moistest 10% and driest 10% of sondes in Fig. 6b are much more similar to those for day 70 of the *ch* simulation than for day 70 of the *sq* simulation in Fig. 4b; indeed, the extreme 10% quantiles in the *sq* simulation at day 70 within the lower and middle free troposphere are much more extreme than even the extreme 1% quantiles for the sondes. The driest 10% quantile in the *sq* simulation at day 70 suggests that air is subsiding from the upper troposphere down to almost 900 hPa without encountering significant moistening by mixing or convection, something not seen in the observations.

Figure 6c, d shows similar analysis to Fig. 6a, b but for relative humidity (defined with respect to ice for temperatures below 0$^{\circ }$C) and CRH. Note that, even for the driest 1% of sondes, relative humidity in the boundary layer is always above 65% on average. The corresponding profiles for the simulations in Fig. 4c, d are consistent with the *ch* simulation being more realistic than the *sq* simulation, at least regarding humidity variability. Note that the near-surface relative humidity averaged for the driest 10% of the domain in the *sq* simulation at day 70 is about 10% drier (in relative humidity units) than the average for the driest 1% of sondes at Nauru.

Table 1 compares surface observations of relative humidity at Nauru with relative humidity at the lowest model level (37 m) in the *ch* simulation from Wing and Cronin (2016). The values for most of the percentiles are comparable, except the simulation has a much lower minimum value than the Nauru observations (32.5% compared to 52.0%).

**Table 1:** Values of surface relative humidity (%) at Nauru (averaged from station data over 1 h centered around on each sonde launch time) and lowest model level (37 m) relative humidity (%) in the 305 K Channel simulation from Wing and Cronin (2016)

Percentile	Nauru	Channel simulation
Minimum	52.0	32.5
1st	57.9	58.6
25th	70.8	69.9
50th	76.8	73.5
75th	82.3	77.5
99th	92.8	93.0

The statistics from the channel simulation are computed over the final 25 days of that simulation

**Fig. 7:**
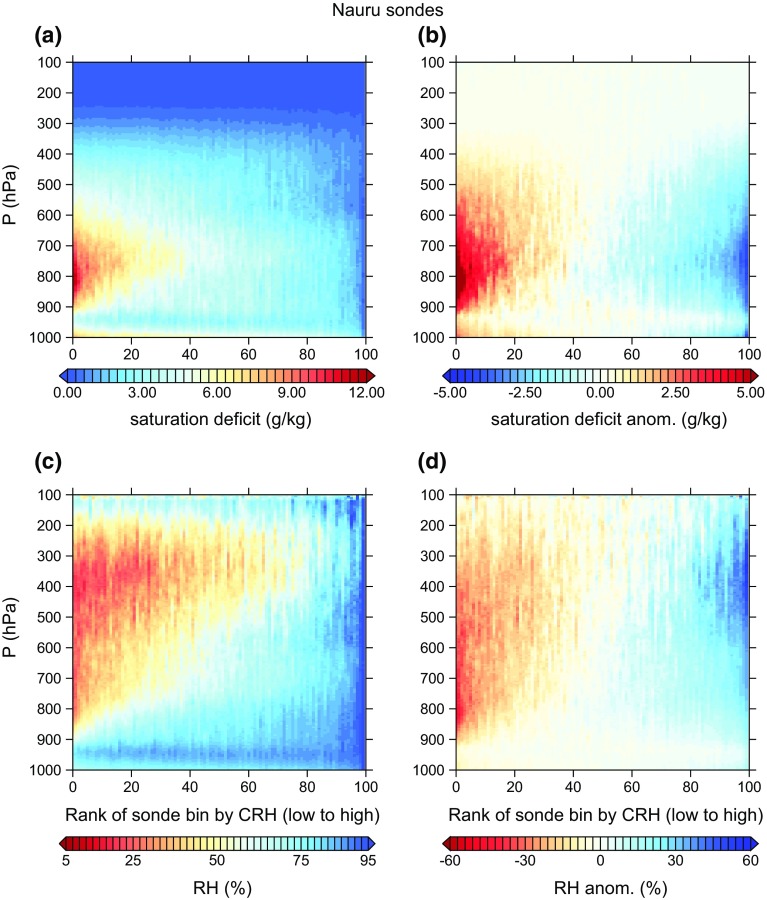
Nauru radiosondes: **a** saturation deficit $(\hbox {g}\,\hbox {kg}^{-1})$ for all sondes ranked by CRH and averaged in 100 equally populated bins, **b** anomaly of each bin in **a** from the all-sonde mean saturation deficit at each level, **c** as in **a** but for relative humidity (%), and **d** as in **c** but for relative humidity

**Fig. 8:**
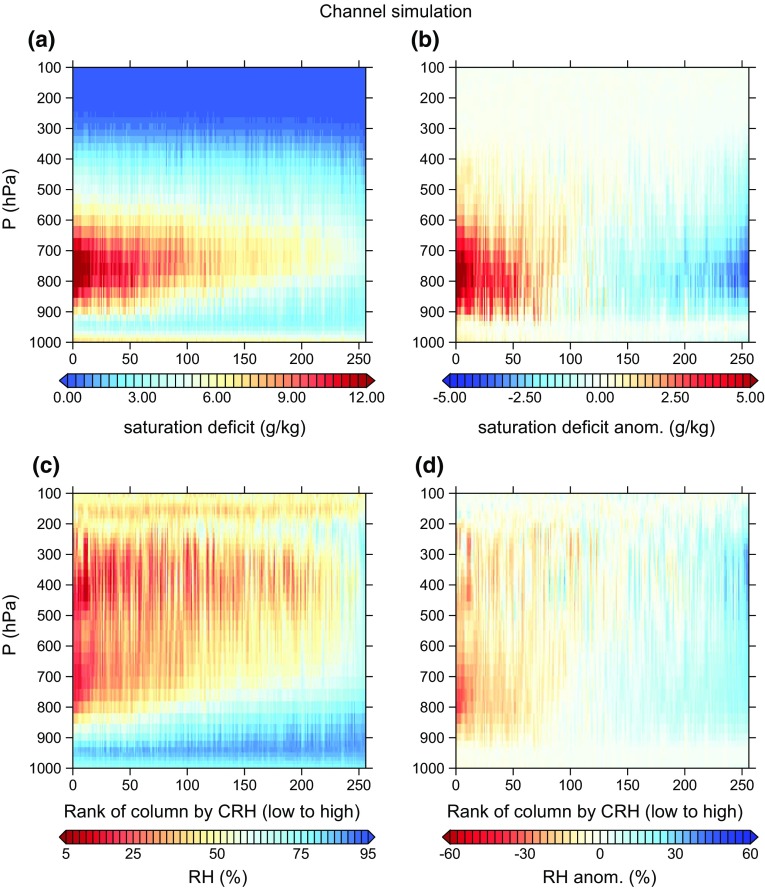
As in Fig. 7 but for channel simulation at 305 K from Wing and Cronin (2016). **a** Saturation deficit $(\hbox {g}\,\hbox {kg}^{-1})$ for all sondes averaged over 192 km $\times$ 48 km blocks and ranked by block-averaged CRH, **b** anomaly of each block in **a** from the domain-mean saturation deficit at each level, **c** as in **a** but for relative humidity (%), and **d** as in **c** but for relative humidity

Figure 7 shows contour plots of all 3491 sondes ranked by CRH and divided into 100 equally populated bins. These show both relative humidity and saturation deficit (saturation specific humidity minus specific humidity). A similar plot for the *ch* simulation from Wing and Cronin (2016) is shown in Fig. 8, and Fig. 7d can be additionally compared with a similar figure from day 90 of the square simulation at 305 K SST in Wing and Emanuel (2014, their Fig. 11). That figure shows that the square simulation has a large spread in relative humidity between 1 and 2 km height of about 100% between moist and dry regions, while the Nauru sondes show a spread of 60% at most in that layer and a much larger number of bins with small anomalies. The channel simulation (Fig. 8), on the other hand, is much more comparable to the Nauru sondes, indicating that this simulation has realistic humidity variability.

Radiosondes from other tropical locations, such as the Bay of Bengal and the eastern tropical Pacific, also reveal significant variability in mid- and upper-tropospheric relative humidity and little variation in boundary layer moisture (e.g., Zuidema et al. 2006; Zuidema and Mapes 2008), though these locations are subject to large-scale circulations that can bring remote influences from neighboring landmasses. Radiosondes from the equatorial Indian Ocean also demonstrate that most of the relative humidity variability is contained within the middle troposphere (Johnson and Ciesielski 2013), where the signature of self-aggregation may be first detected (Mapes 2016).

There are reasons to expect that the Nauru sondes and tropical sondes from these other locations would not necessarily look exactly like idealized self-aggregation simulations (or indeed, would not be representative of tropical maritime observations more generally). For instance, these sondes are generally launched from islands, which could have local effects on convection, and transport of air from landmasses or higher latitudes could also cause differences compared with idealized conditions. Additionally, we may not necessarily think of idealized aggregated convection as something that would or should be representative of typical tropical conditions anyway.

However, since humidity variability is fundamentally linked to both the contributing processes and large-scale impacts of self-aggregation, it is important to consider possible reasons why the channel simulation appears to have more realistic humidity variability, while the square simulation is too extreme. One possibility is that the channel simulation is “getting the right answer for the wrong reason”, for instance because its quasi-2D geometry leads to a spurious strong wind shear similar to that found in 2D simulations in Held et al. (1993). Although the channel simulation does have tropospheric along-channel mean wind and vertical wind shear that are larger than values in the square simulation, the channel values are of order $1\,\hbox {m}\,\hbox {s}^{-1}$ for both quantities, and this is not overly strong compared with typical tropical mean values. The channel simulation is 192 km wide, which allows for multiple convective systems and associated cold pools to exist and propagate along the shorter dimension. Subsidence in the driest regions is actually stronger in the channel simulation relative to the square simulation, though ascent in the moist regions is weaker. While determining the reasons for the differences between the simulations is beyond the scope of this paper, it is likely that the channel simulation has more mixing and transport between convective and subsidence regions—animations (not shown) reveal that boundaries between convective and subsidence regions are less stationary, and closer to the center of subsidence regions, in the channel simulation.

While the above discussion does not definitively endorse one model domain geometry over another, this type of analysis is informative in starting to address the extent to which idealized aggregated convection is similar to organized convection in the real world, and we hope that it helps frame future comparisons with other data. For instance, analysis tracing air particles back to their time of last condensation within both a modeling construct and observations could be helpful (e.g., Pierrehumbert 1998), as well as a spectral analysis to determine if key time scales are matched within both.

### Equatorial Wave Dynamics

Earth’s latitudinally varying rotational effects on large-scale horizontal motions result in equatorial wave dynamics which help shape tropical convective organization. For instance, the MJO interacts with equatorially trapped moist Kelvin and Rossby waves, and these dynamics are also important for the development of the Hadley Circulation, the inter-tropical convergence zone (ITCZ), and monsoons. While self-aggregation is generally defined not to include the effects of a latitudinally varying Coriolis parameter, a few studies have looked for the processes that lead to self-aggregation in simulations that do include such effects. Bretherton and Khairoutdinov (2015) found that radiative feedbacks were likely to be important mainly for large-scale convective organization in their near-global RCE channel runs. Arnold and Randall (2015) performed global aquaplanet simulations (using a superparameterization setup in which 2D CRMs are embedded in each large-scale model grid cell) with uniform SST both with and without rotation and found similarities in diabatic feedbacks between the self-aggregation in the non-rotating setup and the MJO in the rotating setup. Holloway (2017) found that simulations of real near-equatorial case studies using a limited-area CRM setup also showed similarities to idealized self-aggregation, although the effects of suppressing interactive radiation were constrained by the imposed lateral boundary conditions. More studies are needed to probe links between self-aggregation and convective organization that interacts with equatorial wave dynamics.

### Ocean Interaction and Feedback

Nearly all studies of self-aggregation have used atmosphere-only simulations. However, there are a few studies that have used coupled models, and they generally find that ocean coupling slows or prevents self-aggregation. For instance, an interactive slab ocean experiment slowed down self-aggregation in Bretherton et al. (2005), possibly because of cloud shading. That experiment had a $60\,\hbox {W}\,\hbox {m}^{-2}$ imposed ocean cooling to represent large-scale ocean or atmospheric transport, and after aggregation the SST cooled rapidly due to increased longwave cooling. Khairoutdinov and Emanuel (2010) used a 2-m slab ocean but homogenized the SST horizontally at each time step (thus removing effects like cloud shading) and also found that SST dropped after aggregation occurred. They noted hysteresis, since cooler SSTs could still maintain aggregation that had already been present but could not sustain self-aggregation from homogeneous conditions. Popke et al. (2013) performed global-tropics RCE runs using parameterized convection (with no rotation and homogeneous solar forcing) coupled to a slab ocean and found that large convective clusters formed along with transient SST anomalies. Reed et al. (2015) performed similar global-tropics RCE runs and found that, although ocean coupling slows aggregation compared to runs with fixed warm SSTs (302 K) in agreement with other studies, runs with fixed cool SSTs (as low as 295 K) result in much less organization than runs with similar SSTs and an interactive slab ocean, suggesting a possible link between ocean coupling and the sensitivity of aggregation to SST.

Coppin and Bony (2017) also ran global-tropics RCE simulations without rotation and coupled to a slab ocean and found that the coupled RCE system exhibits some internal variability, arising from the interplay between SST, SST gradients and aggregation. The time scale of this variability depends on the depth of the ocean mixed layer, and for a large range of depths, it occurs at the interannual time scale, suggesting a possible link to internal modes of variability in the real tropical ocean atmosphere system such as El Niño Southern Oscillation (ENSO). They also showed that, at this time scale, the relationship between SST and aggregation could be very different from (or even opposite to) that found in prescribed SST simulations or in coupled RCE simulations on long time scales.

Hohenegger and Stevens (2016) ran high-resolution coupled RCE runs (without imposed ocean cooling, but with reduced solar insolation equivalent to that averaged over the full Earth rather than the tropics) and found that aggregation seemed to prevent a runaway greenhouse effect, providing “radiator fins” to the idealized climate in the dry subsiding regions analogous to the role of the subtropics proposed by Pierrehumbert (1995). They also found that slab oceans with small depths can slow or prevent self-aggregation, similar to studies mentioned above. This delay stems from the development of SST gradients which cause a low-level circulation opposing the one that favors self-aggregation. Furthermore, Hohenegger and Stevens (2016) suggest that cloud feedbacks and resulting aggregation and coupled equilibrium states are very different at high resolution using explicit convection versus similar runs using parameterized convection from Popke et al. (2013), showing another example of model disagreement with regards to these processes.

While atmosphere–ocean coupling has been extensively studied for large-scale tropical convective phenomena such as the MJO (cf. DeMott et al. 2015), observational work is needed to explore the interactions between organized tropical convective systems and SST or sea surface salinity across scales. Specifically, this analysis could look at processes important for aggregation in idealized models.

## Observational Perspectives on Aggregation in a Warming Climate

Several modeling studies suggest that convective aggregation depends on surface temperature, although the exact nature of this dependence remains uncertain. The initiation of aggregation is found to occur more easily at certain temperatures (Coppin and Bony 2015), particularly when considering a given domain size (Wing and Emanuel 2014). Once initiated, the clumping of aggregation in some studies tends to strengthen as the surface temperature rises (Coppin and Bony 2015), though other studies find that the degree of aggregation is relatively insensitive to SST (Wing and Cronin 2016; Holloway and Woolnough 2016; Hohenegger and Stevens 2016). Several interpretations have been proposed for the temperature dependence of the initiation mechanisms (Sect. 2.3). Some of them invoke the nonlinearity of the Clausius–Clapeyron relationship, the sensitivity of the clear-sky longwave radiative cooling of the atmospheric column to lower-tropospheric longwave opacity (Emanuel et al. 2014), or the sensitivity of the low-cloud cover to temperature (e.g., Coppin and Bony 2015; Wing and Cronin 2016; Holloway and Woolnough 2016), and these temperature dependences differ across models. On the other hand, the interaction between temperature, high-cloud radiative effects, and dynamics has been proposed by Bony et al. (2016) as a mechanism for stronger clumping of convection at the aggregated equilibrium state over warmer surfaces. That study argues that, owing to the dependence of static stability on temperature and pressure, as the climate warms anvil clouds not only rise to a higher altitude but also shrink in horizontal area. This behavior, referred to as the “stability-iris” effect in Bony et al. (2016), concentrates the atmospheric cloud radiative effects of anvil clouds and enhances the horizontal gradients in atmospheric radiative cooling (enhancing the cooling in subsiding areas and reducing it in convective areas), which could lead to enhanced convective aggregation.

Given the implications that a dependence of convective aggregation on temperature may have for climate (Sect. 1.1), it is important to verify whether this dependence seen in some models is confirmed by observations. However, very few studies have investigated this issue so far. Long time series of convective aggregation indices have now been produced (e.g., Tobin et al. 2012; Tan et al. 2015), but they have not been analyzed in this perspective yet.

What has been investigated, on the other hand, is the temperature dependence of various large-scale organized convective phenomena that share many characteristics with convective aggregation in idealized models. One of these is the MJO, which likely represents a very large-scale manifestation of convective aggregation in the tropics (Khairoutdinov and Emanuel 2010; Arnold and Randall 2015). There is modeling evidence that MJO activity increases when the climate is warming (e.g., Caballero and Huber 2010; Arnold et al. 2015), though MJO-like behavior has been found even at temperatures as cold as $1\,^{\circ }\hbox {C}$ (Pritchard and Yang 2016); this finding of increased MJO activity with increased SST in models is qualitatively consistent with observations that suggest linear increases in the intensity and number of MJO events over the last 50 years (Jones and Carvalho 2006).

Tropical cyclones likely constitute another spectacular manifestation of convective aggregation. But unfortunately, no such consistency has yet been reached between their modeled and observed behavior with temperature. Idealized RCE simulations performed in a rotating framework suggest that the number of tropical cyclones decreases as surface temperature rises, while their intensity and precipitation rate increases (Nolan et al. 2007; Held and Zhao 2008; Khairoutdinov and Emanuel 2013). Climate projections made with general circulation models also suggest such a tendency, although the relationship between tropical cyclones and temperature very much depends on the metrics used for warming (Knutson et al. 2013). On the observational side, however, trends in tropical cyclones and their relationship to temperature remain elusive (Stocker et al. 2013). This is partly due to the limited availability and quality of long-term historical records, but also to the large number of global and regional factors that influence the occurrence and intensity of tropical cyclones. In particular, it is difficult to disentangle a trend associated with global warming from records which are either too short or associated with an insufficient geographical sampling. Another source of complication stems from the fact that tropical cyclone activity does not only depend on absolute surface temperature: it is also affected by factors such as the temperature difference between the surface and the tropopause (Emanuel 1987), the local surface temperature relative to the tropical mean (Lin et al. 2015), the wind shear and mid-tropospheric humidity (Tang and Emanuel 2010), and the upper ocean stratification (Emanuel 2015), and these factors are strongly modulated by the decadal to multi-decadal natural climate variability.

**Fig. 9:**
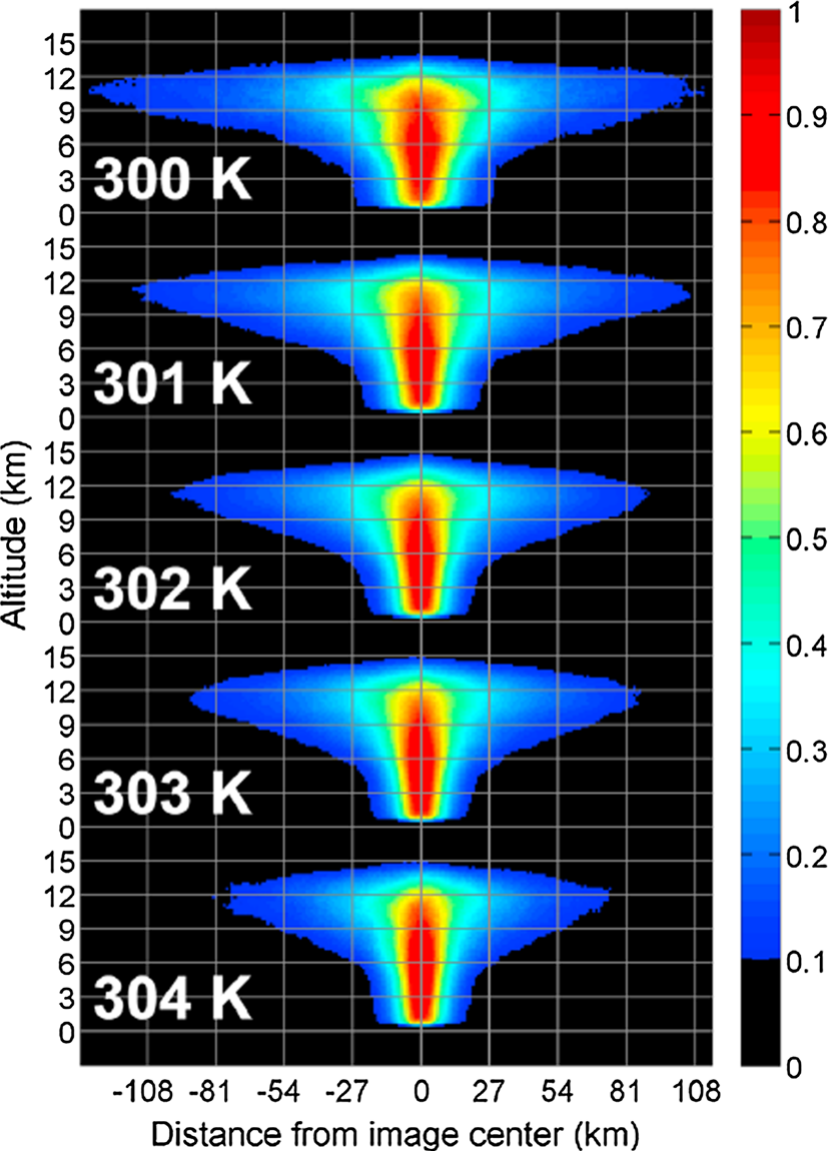
Composites of anvil cloud objects derived from CloudSat observations: the width of anvil clouds is found to decrease as surface temperature increases. From Igel et al. (2014)

To confirm or refute modeling inferences regarding the temperature dependence of convective aggregation, another approach consists of using observations to test the physical processes that contribute to this dependence in models. One such process is the reduction of the anvil cloud amount as the climate warms (Bony et al. 2016). Some observational studies suggest that, on average over the tropics, the anvil cloud amount decreases as the surface temperature increases (Zelinka and Hartmann 2011; Igel et al. 2014) as shown in Fig. 9 reproduced from Igel et al. (2014), but other studies do not find strong evidence for such a relationship (Stein et al. 2017). These differences may result from methodological differences: in contrast with other studies, Stein et al. (2017) consider the dependence of anvil cloud amounts on surface temperature for given precipitation and large-scale forcings, which amounts to comparing situations having a fairly similar convective mass flux, and therefore a weaker change in anvil cloud amount with temperature. Also, Stein et al. (2017) and Igel et al. (2014) are only comparing local SSTs colocated with specific cloud scenes, whereas the reduction of anvil cloud amount with warming may be more sensitive to the tropical mean SST, which is the metric used in Zelinka and Hartmann (2011). And Igel et al. (2014) study anvil cloud per individual cloud object, not total anvil fraction, but Stein et al. (2017) find that SCAI values increase with SST, meaning that there are more (and smaller) anvil cloud clusters for warmer SSTs, which could cancel out effects of smaller anvil size per cluster. Additional methodological differences may also contribute to these conflicting results, and they will have to be clarified.

More generally, several methodological issues complicate attempts to investigate the relationship between temperature and convective aggregation in observations. First, unlike idealized modeling studies forced by uniform boundary conditions, the Earth’s climate is associated with gradients in surface temperature which strongly influence large-scale vertical motions in the tropics. As is widely recognized, at the regional scale clouds and convection are much more influenced by the large-scale atmospheric circulation than by local surface temperature (Hartmann and Michelsen 1993; Bony et al. 1997). For this reason, relationships between convective aggregation and surface temperature derived from regional investigations do not necessarily reflect an intrinsic dependence of aggregation on temperature. Second, there is abundant evidence that the relationship between temperature and water vapor or clouds can differ on short versus long time scales (e.g., Dessler 2010). Relationships inferred from observed climate variations on seasonal or interannual time scales may thus differ from the temperature dependence of convective aggregation on decadal time scales and under long-term climate change. Recent results suggest that this might also be the case for coupled RCE simulations (Coppin and Bony 2017), although the extent to which this result applies to other models remains unknown. Finally, models suggest that convective aggregation can behave very differently in cold and warm climates. For instance, Abbot (2014) predicted stronger convective aggregation on a Snowball Earth than in the modern climate, but observations of clouds and convection are only available for a limited time period and thus for a very narrow range of surface temperatures. To explore possible changes in convective aggregation in more drastically different ranges of temperature, one must consider paleoclimatic changes using proxy data. Techniques of paleotempestology offer opportunities to reconstruct tropical cyclone activity at different periods of the past and for a range of time scales (e.g., Liu and Fearn 2000; Donnelly and Woodruff 2007) and could be very useful for this purpose. The isotopic composition of water is very sensitive to the organization of convection (e.g., Lawrence et al. 2004; Risi et al. 2008), and therefore long-term isotopic records (as well as recent satellite observations of water isotopes) could be used to explore changes in convective organization with climate.

## Future Observational Aspirations

Much remains to be done in terms of observing convective aggregation. Using satellite observations, the variability of aggregation at different time scales could be investigated, as well as its relationship to local and remote surface and atmospheric conditions. Besides this, it would be nice to investigate whether the physical mechanisms found to play a role in the initiation of aggregation in models can also be observed in nature. For instance, is there evidence for the formation of radiatively driven cold pools in the dry areas of the tropics? Will future space missions such as the ADM-Aeolus wind lidar mission (Reitebuch 2012) help observe the interplay between low clouds and shallow circulations in the vicinity of deep convection? Will they help us observe radiatively driven cold pools? Can we observe signs of convective self-aggregation? To address these questions, one may analyze observations from field experiments such as those collected during AMIE/DYNAMO in the Indian ocean (Feng et al. 2015) or in the tropical Atlantic as part of the NARVAL-EUREC$^4$A campaigns (Stevens et al. 2015; Bony et al. this issue). One may also think of organizing a field campaign specially dedicated to these questions.

The ISSI workshop in February 2016 on “Shallow clouds and water vapor, circulation and climate sensitivity” brought together scientists using numerical simulations to study convective organization with scientists at the forefront of observational work, including experts on remote sensing of clouds and their environment. In this section, we present some perspectives on novel approaches to using satellite data to observe convective aggregation and the processes discussed above. Another paper (Lebsock this issue) also presents some promising new work along these lines using CloudSat and other A-Train satellites, complementing work by Stein et al. (2017). We also propose another possible way forward using a ground-based observational network.

### Evolution of Convective Organization Using Satellite Data

The physical processes of convective self-aggregation involve a range of elements from the dynamics of convective systems to the thermodynamics of their rain-free environment. In this section, we review recent work with unique ideas of exploiting the existing satellite capability to study precipitating cloud systems and the surrounding atmosphere. The potential utility of such satellite observations in addressing different aspects of convective self-aggregation is also discussed.

The variability in the large-scale atmospheric state associated with a life cycle of convective systems has been examined with a suite of satellite measurements by Masunaga (2012) and several subsequent papers. Since the sporadic nature of low-Earth orbiting (LEO) satellite overpasses with high-inclination orbits makes it difficult to continuously monitor subdaily scale variations, the variability is statistically reproduced by projecting a large number of snapshots obtained from multiple LEO satellites onto a composite time series. For instance, temperature and humidity profiles from the Atmospheric Infrared Sounder (AIRS) aboard the Aqua satellite are combined with the Tropical Rainfall Measuring Mission (TRMM) Precipitation Radar (PR) so that the evolution of the ambient sounding is constructed over the hours before and after convection develops (Masunaga 2012).

Masunaga (2013) applied water and heat budget analysis to this composite time series. The moisture and MSE (denoted by *h*) budget equations integrated vertically over the troposphere are:

1 $$\begin{aligned} \frac{\partial }{\partial t} \langle q \rangle + \langle \overline{ \nabla \cdot q \mathbf {v} } \rangle = \overline{E} - \overline{P} \end{aligned}$$

and

2 $$\begin{aligned} \frac{\partial }{\partial t} \langle h \rangle + \langle \overline{ \nabla \cdot h \mathbf {v} } \rangle = \overline{S} + L_{\rm v} \overline{E} + \langle Q_{\rm R} \rangle , \end{aligned}$$

where $\langle \cdots \rangle$ designates the vertical integral over the whole troposphere, the overbar denotes horizontal averaging over a large-scale [*O*(100 km)] domain, *q* is specific humidity, $\mathbf {v}$ is horizontal wind, *E* is surface evaporation, *P* is surface precipitation, *S* is surface sensible heat flux, $L_{\rm v}$ is the latent heat of vaporization, and $Q_{\rm R}$ is the radiative heating rate. Each term on the rhs of (1) and (2) is available from satellite observations and the tendency term on the lhs is evaluated from the composite time series, leaving as the only unknowns the second term of (1) and (2), that is, the horizontal convergence of moisture and MSE convergence. The vertically integrated moisture and MSE convergences, although not directly measurable from satellites, are instead derived as the residual in the budget equations. Note that the quantities calculated for these equations could also be used to calculate the diabatic terms of the MSE spatial variance budget from Wing and Emanuel (2014) as suggested in Sect. 2.4.

**Fig. 10:**
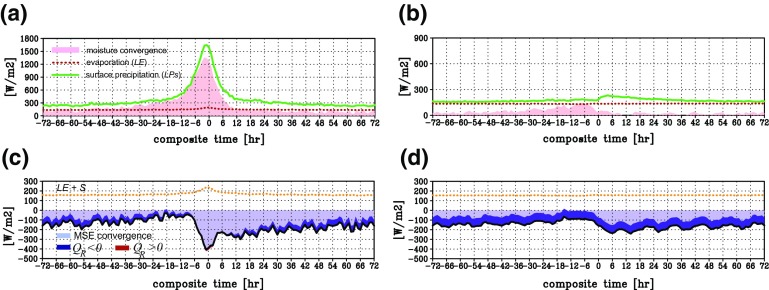
Satellite-derived moisture and MSE budget parameters in composite time series associated with the development and dissipation of convection. **a** Moisture convergence (*shaded*), surface precipitation (*solid line*), and surface evaporation (*dotted line*) for the organized system regime, **b** as in **a** but for the isolated cumulus regime, **c** MSE convergence (*light-shaded*), radiative heating (*heavy shaded*; *red* where positive and *blue* where negative) on the top of MSE convergence, surface heat flux (*dotted line*) for the organized system regime. **d** As in **c** but for the isolated cumulus regime. All parameters including precipitation and evaporation are plotted in energy flux units $(\hbox {W}\,\hbox {m}^{-2})$

Figure 10 shows the composite evolution of different budget terms for both the “organized system” and “isolated cumulus” regimes. These two regimes are separated by applying different thresholds to the areal coverage of TRMM-detected precipitation cells (i.e., <25% for isolated cumuli and >50% for organized systems), aimed at delineating the elements of atmospheric thermodynamics that are favorable or unfavorable for convective organization (Masunaga 2014). The primary moisture source of precipitation is moisture convergence during hours around the peak convection in the organized system regime (Fig. 10a), while precipitation nearly balances out the local moisture supply from surface evaporation in the isolated cumulus regime (Fig. 10b). In both the regimes, evaporation stays almost constant over time at $\sim$100–150 W m$^{-2}$, which suffices to produce modest rainfall from isolated cumuli but needs to be supplemented by a large dynamically driven import of moisture to feed organized systems.

The dynamics specific to organized convective systems is illustrated in light of the MSE budget (Fig. 10c), where MSE convergence stays overall negative but nearly vanishes to zero as convection intensifies (discussed in detail by Masunaga and L’Ecuyer 2014). The zero MSE convergence, or neutral gross moist stability (GMS), implies that the import of moisture is just large enough to drive the large-scale adiabatic ascent and hence allows a self-sustaining growth of convection (Masunaga 2014). In the isolated cumulus regime (Fig. 10d), MSE convergence vanishes as in the organized system regime but the enhanced radiative cooling, owing to reduced high clouds, appears to work against the further growth of convection that could otherwise occur.

Note that the composite time series above are not to be interpreted as convective self-aggregation itself being in progress. Idealized simulations demonstrate that convective self-aggregation proceeds over a week or two (Tompkins 2001) or a few months (Bretherton et al. 2005; Wing and Emanuel 2014), which is a time scale substantially longer than the life cycle of individual convective systems (a few days at most) as depicted in Fig. 10. The isolated cumulus regime and organized system regime, if put into the context of self-aggregation, may be each a representation of the states before and after the self-aggregation takes place (or outside and inside the area of aggregated convection). From this perspective, the convective self-aggregation could be considered as a “phase transition” from the isolated cumulus regime to the organized system regime. Figure 10c, d suggests that a key role in the transition, if it occurs, would be the magnitude of radiative cooling, which is in line with idealized simulations (Muller and Bony 2015) and theories of convective self-aggregation (Emanuel et al. 2014). This hypothesis may be tested by separating the composite analysis among different degrees of convective aggregation using, for example, SCAI (Tobin et al. 2012). With the other environmental conditions such as SST being equal, a set of composite time series constructed with different SCAI values would provide an observational test bed to examine the self-aggregation processes in the context of moisture and thermal budgets. This would be an interesting line of research to pursue in the future.

### Spaceborne Cloud Radar Approaches

Novel analyses of newer satellite assets that have not traditionally been applied to study convection may offer potential for advancing our understanding of the coupled radiative and hydrological responses to convective aggregation. There is growing acceptance of the utility of spaceborne cloud radars, in particular, for characterizing the distribution, internal structure, spatial organization, updraft intensity, and radiative environments of convection. New methods for discriminating precipitating scenes, isolating convective cores, and profiling radiative fluxes and heating rates both within cloud and in the adjacent cloud-free pixels are becoming sufficiently mature to shed new light on the coupled energy and water cycle impacts of convective aggregation (Haynes et al. 2009; Lebsock and L’Ecuyer 2011; Henderson et al. 2013; Matus and L’Ecuyer 2017).

Igel and Heever (2015), for example, used CloudSat observations to establish a quantitative link between the area of convective anvils and the associated convective cores. Unlike previous studies that relied on coarser or less direct methods for identifying convective updrafts, the high sensitivity and relatively high spatial resolution of the CloudSat Cloud Profiling Radar (CPR) provide an unambiguous means of discriminating precipitating and non-precipitating pixels with associated ice cloud area and vertical structure from a single sensor. Examining nearly 5 years of CloudSat observations over the tropical oceans, Igel et al. (2014) and Igel and Heever (2015) demonstrate that anvil widths systematically decrease while anvil temperatures become colder with increasing SST, as discussed in Sect. 4. In addition, the width of associated cloud object pedestals (the cloud shapes at the base of the anvils) decreases with increasing SST. These findings could be consistent with a trend toward more aggregated convection over warmer oceans, though it should be noted that, as discussed in Sect. 4, these studies look only at anvil size per cloud object, not at total cloud area or how individual cloud objects are spatially distributed. Furthermore, these studies do not explicitly control for precipitation intensity or divide observations into different large-scale circulation regimes, and they look at local SST rather than tropics-wide SST. Stein et al. (2017) use CloudSat-CALIPSO data to link cloud amount to aggregation, showing larger areas of anvil cloud and less low cloud in regions with less large-scale aggregation for a given large-scale rain rate, although they find less dependence of anvil fraction on (local) SST as discussed in Sect. 4.

The greatest potential of cloud radar observations for advancing theories of convective aggregation may, however, reside in recent efforts to infer internal dynamics and related processes (Luo et al. 2010; Nelson et al. 2016). As convection evolves to a more aggregated state, there is reason to anticipate that convective buoyancy and entrainment rates will change owing to the reduced convective area and cloud lateral boundaries. Luo et al. (2010) used the difference between cloud top temperature (CTT) and that of the ambient environment at the radar-defined cloud top height (CTH) to estimate convective buoyancy and entrainment rates in individual convective systems. CloudSat reflectivity observations effectively remove the ambiguity between cloud top temperature and height, allowing buoyancy to be estimated by comparing the observed CTT to the temperature at the CTH in the environmental sounding. Entrainment rates are then estimated through iterative application of an entraining plume model to obtain the best match with observed storm vertical structure. Luo et al. (2010) paint a familiar picture of tri-modal tropical convection made up of shallow, mid-level congestus, and deep convective modes (e.g., Johnson et al. 1999) but further characterize the composite dynamic processes within each mode. Nearly all deep convection has negatively buoyant cloud tops and smaller entrainment rates while congestus can be separated into distinct “transient” and “terminal” modes with positive and negative buoyancy (smaller and larger entrainment rates), respectively.

Given the challenges associated with directly observing the time evolution of convective cloud structures on the scales required to observe convective aggregation, it may be argued that composites of such observation-based estimates of dynamic and thermodynamic processes will be key to testing model-based inferences regarding the driving processes. It is very likely, for example, that transitions from scattered to aggregated states of convection will be accompanied by a shift in the relative frequencies of convective states, leading to corresponding changes in domain-mean buoyancy and entrainment rates that may be measured through a similar approach. While Luo et al. (2010) do not characterize the properties of shallow convection, recent work has demonstrated that evaporation and condensation rates in shallow convection can also be inferred from cloud radar observations, offering the potential to further address the role of shallow convection in the transition from isolated to aggregated convection (Nelson et al. 2016).

Spaceborne cloud radar observations also offer potential for testing hypothesized feedbacks and energy and water cycle impacts of convective aggregation. Luo et al. (2014) use time-differenced infrared brightness temperatures to relate cloud top vertical velocities to convective mass transport and precipitation efficiency, two central physical characteristics linking the causes and effects of convective aggregation. They demonstrate that stronger updrafts correlate with higher precipitation echo-tops, increased convective mass fluxes, and heavier rainfall throughout the tropics. While these studies do not definitively test emerging theories concerning convective aggregation, they attest to the maturity of novel process-related datasets from cloud radar observations and suggest that pursuing new ways of integrating spaceborne cloud radar into future studies of convective aggregation is warranted.

### Feasibility of a Ground-Based Observational Network

Simulations of convective aggregation have shown that there is a marked difference in the water vapor profiles in the dry and moist regions (Fig. 4), and the longwave radiative heating difference between the two regions (Fig. 5) results in an up-gradient flow just above the boundary layer that works to further enhance this moisture gradient (Sect. 2). This characteristic difference between dry and moist regions is an important indicator of aggregation, and therefore something that a field experiment could target. In this section, we discuss the feasibility of such an experiment (for instance as part of a field campaign) using currently available ground-based instruments.

**Fig. 11:**
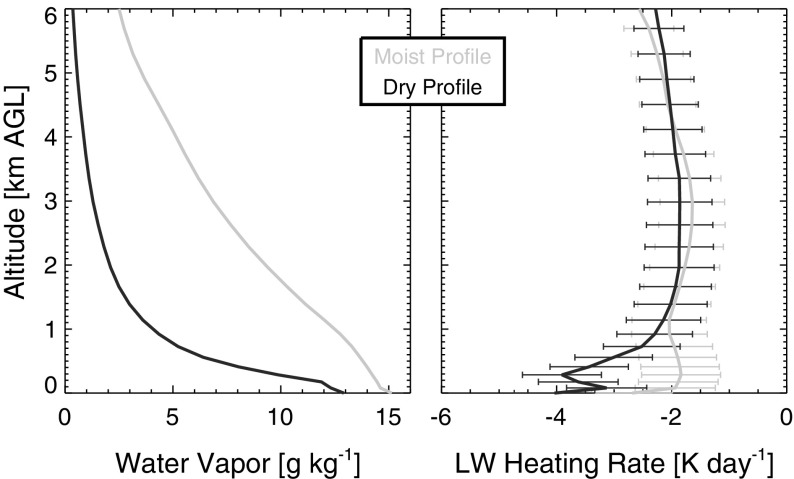
Water vapor mixing ratio profiles from a dry (*black*) and moist (*gray*) region of an RCE simulation (at day 30) where convective aggregation occurred (*left*), and the corresponding longwave radiative heating rate profiles computed using the RRTM (*right*). The *error bars* on the heating rate profiles were computed by propagating the uncertainties in the Atmospheric Emitted Radiance Interferometer (AERI) retrieved profiles through the RRTM

An example of water vapor profiles from the dry and moist regions in a simulation of aggregation from Muller and Bony (2015), along with longwave radiative heating rates in cloud-free scenes computed using a radiative transfer model (RRTM; Mlawer et al. 1997), are shown in Fig. 11. The differences in the shape of the water vapor profiles result in an extra 2 K day$^{-1}$ clear-sky longwave cooling in the boundary layer. This boundary layer cooling will be enhanced if there are shallow liquid water clouds at the top of the boundary layer; cumulus are often seen in the dry regions of simulations that show aggregation.

The challenge of any field experiment that aims to investigate the results shown by numerical simulations of convective aggregation is the ability to observe water vapor profiles, especially in the boundary layer, with the needed accuracy to yield significant differences in the computed radiative heating rate profiles (cf. Stevens et al. this issue). Many different boundary layer thermodynamic profiling technologies are currently being used; Wulfmeyer et al. (2015) provides a review of these instruments. Satellite sensors have difficulty observing the thermodynamic structure of the boundary layer, especially if there are clouds in the scene; the limitations of satellite observations of water vapor also affect reanalyses (Pincus et al. this issue). Thus, a network of multiple ground-based remote sensors distributed over some area is the best option if a long duration dataset is desired to observe the processes that lead to convective aggregation.

Of ground-based sensors, active remote sensors like water vapor Raman lidar and differential absorption lidar (DIAL) have a special appeal because of their vertical resolution and accuracy. However, there are no commercially available water vapor Raman lidars or DIALs, and thus any network of lidars would consist of systems from multiple research groups where each lidar would have its own sensitivity and uncertainties that may make the analysis of a network of these datasets more challenging. However, there are commercially available microwave radiometers and infrared spectrometers, and thus a network composed only of one of these types of instrument would be homogeneous and potentially easier to analyze.

Passive remote sensors like microwave radiometers and infrared spectrometers observe radiance, and retrieval algorithms are needed to derive thermodynamic profiles from these observations. Several studies have investigated the accuracy and information content of these retrieved profiles. Löhnert et al. (2009) used an instrument system simulation experiment to demonstrate that infrared spectrometers such as the Atmospheric Emitted Radiance Interferometer (AERI, Knuteson et al. 2004a, b) have 2–4 times more information on both the temperature and water vapor profile than microwave radiometers, which leads to improved accuracy in the AERI-retrieved profiles under clear-sky conditions. Blumberg et al. (2015) and Weckwerth et al. (2016) both confirmed that the AERI-retrieved water vapor profile was more accurate than microwave radiometers below cloud base or in cloud-free scenes using real observations.

A natural question is: Do the AERI retrievals have the sensitivity to distinguish between the longwave radiative cooling rate profiles in the dry and moist columns seen in convective aggregation scenarios? If so, then the AERI would be a good choice to include in any ground-based network that is established to study convective aggregation from observations. The AERI retrieval algorithm developed by Turner and Löhnert (2014), which is able to retrieve lower-tropospheric thermodynamic profiles in both clear and cloudy conditions, provides a complete error covariance matrix for each retrieval. Thermodynamic profiles derived from a Monte Carlo sampling of this error covariance matrix were used to derive the RRTM to compute cloud-free longwave radiative heating profiles, and the $1-\sigma$ uncertainties at each level are shown in Fig. 11 (right). This demonstrates that the AERI has the accuracy to determine the radiative heating rate profiles in the two clear-sky scenes. However, in moist convective regions, where clouds are numerous and can dominate radiation fluxes, a combination of AERI and microwave radiometer retrievals may be desirable to ensure sufficiently accurate humidity profiles (Löhnert et al. 2009; Turner and Löhnert 2014). Furthermore, low clouds in the dry region contribute significantly to self-aggregation in numerical simulations, so it would also be desirable to measure vertical profiles of cloud water. This would enable a calculation of the total radiative heating rate profiles. Cloud radars are the only type of instrument capable of this type of measurement, but are likely prohibitively expensive to deploy in a network as proposed here. A first step could be the deployment of ceilometers, which are a standard, relatively inexpensive, autonomous, weak lidar used primarily at airports to determine cloud base height.

A main strength of microwave and AERI measurements is their ability to measure vertical profiles of moisture. If deployed in a network, the profilers in combination also provide spatial context. A further extension integrates the surface-based measurements with a satellite view of the CWV, thereby more fully interrogating the moisture budget expressed in (1) (Hannah et al. 2016). The satellite can also be integrated with satellite-derived perceptions of the precipitation and cloud distribution, while the surface-based network provides further information on low clouds not easily detected from space and fills in measurements in-between satellite overpasses, so that a rich, dense, three-dimensional construction of a moisture field can be constructed that is large enough to encompass both dry and moist regions. A remaining difficulty may be the typically short time spans for a field deployment, muddying an interpretation of self-aggregation from data. Nevertheless, high-resolution large-domain simulations coincident with such field observations, and combined with observed surface fluxes and top-of-atmosphere radiative fluxes, will inspire a deeper confidence in the theory of self-aggregation derived from RCE simulations and help determine the relative importance of contributing processes.

## Conclusions

Observing convective organization is not a new pursuit, as evidenced by the literature review in this paper. But as we learn more about how convection clumps in idealized models, there are new opportunities to formulate theories of fundamental convective processes and test them (or at least gauge their plausibility) using observations. Models can also be used at more realistic configurations to form a bridge between idealized simulations and observations, and to help us better frame observational studies.

Insights from idealized simulations are already raising many new questions about how the climate interacts with convective organization. But some findings are dependent on model setup or formulation, and the few existing observational studies of aggregation are not completely consistent with each other or with some model findings—we encourage recent efforts to organize an intercomparison of RCE in models over a range of complexities and configurations to help resolve these discrepancies. There is agreement between models and observations that, as convection becomes clumped into fewer moist regions, the subsidence regions become drier, resulting in a drier large-scale mean environment. This drying, and a reduction of upper-tropospheric stratiform cloud, leads to larger OLR and stronger atmospheric cooling. However, how aggregation and its effects interact with local SST on short time scales and with tropics-wide SST on long time scales is still uncertain in both models and observations, as discussed further below.

Initiation processes, such as radiatively driven cold pools and related shallow overturning circulations, are one obvious observational target. Maintenance processes may be even easier to study in observations, since they can be studied in heterogeneous conditions more typical of convection in nature. There are already links between convective self-aggregation processes in models and observed phenomena such as the MJO and tropical cyclones, with feedbacks involving convection, clouds, moisture, radiation, and surface fluxes being important. In fact, the difficulty of global weather and climate models to simulate these phenomena may be related to problems with those models’ ability to simulate aggregation processes, as mentioned in Wing et al. (2017).

Time scales are longer in idealized self-aggregation from homogeneous conditions than typical time scales of observed growth of organized mesoscale convection, but we have argued that this does not mean that idealized processes are not relevant for real organized convection. This is because time scales vary a lot in idealized models, and exponential growth implies shorter effective time scales when starting from already existing organization as is often found in nature. Furthermore, although the real world certainly contains additional processes that can organize or disorganize convection (such as those reliant on coastlines and orography), and these may dominate where they are faster or stronger than self-aggregation processes, these are likely to be concentrated in particular regions and at particular (especially smaller) space and time scales. This means that self-aggregation processes may be favored in other regions and, perhaps, on larger spatial scales, and they are still likely to be relevant for many phenomena and for climate. Feedbacks allowing for the maintenance of idealized aggregation may also be important for maintaining organized convection in nature, since disaggregation time scales are relatively short when longwave radiation feedbacks are turned off in idealized simulations.

Some preliminary new findings show that an idealized simulation with elongated channel geometry has a more realistic representation of atmospheric humidity than a simulation with a square domain, which has too broad distribution of humidity and is too dry in the driest regions when compared with radiosonde records from Nauru. This is an example of how observations may be used to discriminate between different model configurations and address concerns that may otherwise cast doubt on the relevance of aggregation studies in general. Determining the reason for the difference in humidity between these two model configurations is beyond the scope of this paper, and some caution should be exercised when interpreting these results until the reasons and their relationship to physical processes are better understood, since it is possible that the channel simulation gets the “right answer for the wrong reason”. However, possible reasons for these humidity differences include differences in wind speed caused by stronger overturning circulations in the channel simulations, the development of slightly stronger mean wind and wind shear in the channel simulations, or interactions between large-scale circulations and multiple convective regions causing increased proximity and mixing between moist and dry regions.

Recent work has underlined the potential importance of the sensitivity of aggregation processes to SST and climate change. There are exciting new processes being proposed, such as the “stability-iris” effect (Bony et al. 2016) that predicts smaller anvil fractions in a warmer climate. We are gaining an understanding of how organized convection and climate change may interact, but new questions are being raised about the fidelity of our models. The ability to represent both large-scale circulations and convective processes adequately is still a challenge for idealized models, and the effects of more complex processes such as ocean coupling are only beginning to be explored. There are also challenges in using observations of the recent past to test model behavior in both idealized and more realistic simulations of projected future climate. Specifically, we lack long observational records, and studies of regional snapshots or individual cloud elements over short time periods may not scale up to tropics-wide behavior on long time scales. There are also differences in the SST dependence of initiation processes versus maintenance processes in idealized self-aggregation which need to be further explored in both models and observations. But the problem of aggregation in a warming world is an important one and deserves a sustained research effort.

In addition to encouraging the continuing endeavor of confronting self-aggregation processes with observations in general, we have proposed several specific lines of work which appear promising. These include:

- existence, formation, and structure of radiatively driven cold pools
- MSE spatial variance budget analysis
- particle tracing back to last saturation (model and observations)
- radiosonde time scale analysis
- changes in satellite-observed aggregation state and anvil fraction over last few decades (and further investigation of sensitivity to SST)
- paleotempestology using long-term isotopic records
- satellite analysis of link between water vapor isotopes and aggregation
- multiple-satellite temporal evolution of MSE budget for aggregated convection
- spaceborne radar to infer convective processes
- ground-based observational network

While this list is a good start, there are surely other opportunities to further our knowledge of aggregation processes in idealized models using observations. And as the field advances, there will be more ideas to test and explore. We hope the work reviewed and proposed here is the beginning of an exciting new engagement between modeling and observational studies of organized convection.

## Footnotes

1 Mapes and Houze (1993) found that half of the very cold cloudiness was contributed by cloud clusters greater than $2 \times 10^4 \, \text {km}^2$ in size and half of the moderately cold cloudiness was contributed by cloud clusters greater than $10^5 \, \text {km}^2$ in size. Mapes (1993) found that 43% of the total cold cloud coverage in the tropics was associated with large superclusters.

2 Tan et al. (2013) found that the cloud regime associated with organized convection contributes 45% of total tropical rainfall. Nesbitt et al. (2000) found that precipitation features that include an MCS contribute 38–55% of total rainfall in various regions of the tropics.

3 SST = 305 K, no rotation, diurnal cycle of insolation at 19.45 N at perpetual Julian day 80.5, Rapid Radiative Transfer Model (RRTM) radiation scheme, 3 km horizontal resolution, 64 vertical levels, rigid lid at 28 km, doubly periodic lateral boundaries, initialized with white noise in boundary layer temperature field.
